# Comprehensive characterization of protective face coverings made from household fabrics

**DOI:** 10.1371/journal.pone.0244626

**Published:** 2021-01-13

**Authors:** Suvajyoti Guha, Alexander Herman, Ian A. Carr, Daniel Porter, Rucha Natu, Shayna Berman, Matthew R. Myers

**Affiliations:** Division of Applied Mechanics, Office of Science and Engineering Laboratories, Center for Devices and Radiological Health, United States Food and Drug Administration, Silver Spring, MD, United States of America; Indian Institute of Technology Bombay, INDIA

## Abstract

**Background:**

Face coverings constitute an important strategy for containing pandemics, such as COVID-19. Infection from airborne respiratory viruses including Severe Acute Respiratory Syndrome Coronavirus 2 (SARS-CoV-2) can occur in at least three modes; tiny and/or dried aerosols (typically < 1.0 μm) generated through multiple mechanisms including talking, breathing, singing, large droplets (> 0.5 μm) generated during coughing and sneezing, and macro drops transmitted via fomites. While there is a growing number of studies looking at the performance of household materials against some of these situations, to date, there has not been any systematic characterization of household materials against all three modes.

**Methods:**

A three-step methodology was developed and used to characterize the performance of 21 different household materials with various material compositions (e.g. cotton, polyester, polypropylene, cellulose and blends) using submicron sodium chloride aerosols, water droplets, and mucous mimicking macro droplets over an aerosol-droplet size range of ~ 20 nm to 0.6 cm.

**Results:**

Except for one thousand-thread-count cotton, most single-layered materials had filtration efficiencies < 20% for sub-micron solid aerosols. However, several of these materials stopped > 80% of larger droplets, even at sneeze-velocities of up to 1700 cm/s. Three or four layers of the same material, or combination materials, would be required to stop macro droplets from permeating out or into the face covering. Such materials can also be boiled for reuse.

**Conclusion:**

Four layers of loosely knit or woven fabrics independent of the composition (e.g. cotton, polyester, nylon or blends) are likely to be effective source controls. One layer of tightly woven fabrics combined with multiple layers of loosely knit or woven fabrics in addition to being source controls can have sub-micron filtration efficiencies > 40% and may offer some protection to the wearer. However, the pressure drop across such fabrics can be high (> 100 Pa).

## Introduction

Fit tested N95 respirators are known to play a crucial role in offering protection to the adult wearer against airborne pathogens, such as the coronavirus disease 19 (COVID-19) pandemic caused by severe acute respiratory syndrome coronavirus 2 (SARS-CoV-2). However, the limited availability, and logistical challenges to fit-test the entire United States population means that do-it-yourself (DIY) facemasks are often necessary in public health emergencies involving airborne pathogens. The Center for Disease Control and Prevention (CDC) currently recommends donning DIY face-coverings/cloth-masks for children ages 2 years and above [1]. The World Health Organization (WHO) recommends that the following factors be considered in the design of face coverings: number of layers, breathability, water repellence, shape and fit of masks [2,3]. The WHO has also urged the research community to actively engage in research to assess effectiveness of various interventions. This has prompted research on materials and approaches to creating face coverings from household materials, and a rapid increase in publications on material choices [4–8], number of layers [4–6], attachment mechanisms [1,9], performance during coughing [7,10,11], etc.

Transmission of respiratory viruses can happen through at least three primary modes [12,13]. First small submicron (< 1.0 μm) aerosols that can linger in the air for long periods of time [14,15], are generated while talking, breathing, and singing [16,17]. Second, droplets can be generated during coughing and sneezing [18], which get expelled into the air. These droplets can subsequently dry up to create sub-micron droplet nuclei. Third, large nose drops, or splatters can be transmitted through direct contact [12]. When assessing the performance of household materials for use as face coverings, it is important to test them under conditions that reflect all three modes. Unfortunately, to the best of our knowledge, no studies have characterized fabrics in such a comprehensive manner.

Though respiratory virus aerosols in the sub-micron range had received less attention compared to larger droplets, there is growing evidence that these aerosols may be playing a very important role in transmission during the current COVID-19 pandemic [15,19]. Such aerosols may have a half-life of approximately 3 days at ambient conditions [20]. During this time, these viral aerosols may also collide with ions in air, rendering them somewhat similar to conditioned sodium chloride aerosols, such as those used for certifying N95 respirators [21]. Prior studies suggest that this National Institute for Occupational Safety and Health (NIOSH) methodology is conservative and is the most useful predictor of how a respirator or surgical mask would fare against sub-micron aerosols [22]. However, only a few of the filtration studies with household materials have been performed under such worst-case testing [5,6,23]. Differences in protocols across studies, [4,5,8,11,24] and widely ranging filtration efficiencies for similar materials across various labs [4,5,25] make it difficult to interpret results across studies. While surgical masks and fabrics are believed to be source controls [26,27], and are not intended for protecting the wearer, recently, it has been suggested that masks (surgical and face-coverings) can also protect the wearer to some extent by reducing the inoculum of the virus that results in much milder infections [28]. Therefore, when making a face covering, choosing a fabric with some filtration efficiency for submicron aerosols may yield benefits.

In context of larger droplets, filtration studies of household materials were rare prior to COVID-19 [29], but has started to receive increased attention [7,30]. This mode of transmission is important to study because of two reasons: given the larger size, the viral load of droplets can be considerably higher than sub-micron droplets [31], and filtration efficiency reduces with increasing flow velocities [32], such as those occurring in sneezing. It is not well known how fabric materials would fare against sneezing or coughing velocities that can be orders of magnitude higher than breathing velocities [33]. Almost all the studies done using large droplets and at high velocities have been performed using qualitative or quantitative imaging techniques with little attention to aerosol characterization [7,10,30].

The third mode of infection, the contact or fomite route [12,13], has received least attention in context of face coverings. This route can cause infection in at least multiple potential ways: constant coughing or sneezing by an infected individual can lead to accumulation of significant volumes of fluids in the inner layers of the covering that can then permeate to outer layers; or supra large droplets that can transmit via nose drips or coughs, can permeate through different layers of the fabrics, reach the outermost layer, and get to other surfaces through touch. Conversely, such droplets can permeate through the wearer’s face covering when the subject subconsciously touches the face covering after contacting a contaminated surface. Given that the majority of subjects produce < 2 mL (i.e. 0.1 mL/hour) of sputum volume in a 24-hour period [34] with up to 6.38x10^8^ copies of SARS-CoV-2 per mL reported in saliva [35], a single cough or nose drip, even for a material that is hypothetically 99% efficient in stopping pathogens, can allow up to 5.31x10^5^ copies of SARS-CoV-2 to permeate through. At a coughing frequency of roughly 0.5 an hour [36], the total SARS-CoV-2 copies that can permeate through two 99% efficient layers is ~ 2.7x10^3^ copies, which is comparable to infectious doses for respiratory viruses [37]. Most fabrics are unlikely to be so highly efficient, and therefore the number of viruses that can permeate through can potentially be higher. Therefore, studying the permeability of household materials against such macro droplet volumes is necessary.

The objective of this study is two-fold: first, to develop a systematic approach to characterizing the protection offered by household materials against all the three modes described above; second, to use that methodology to identify household materials that can be used to fabricate breathable cloth coverings with decent filtration efficiency. The size range to define aerosols versus droplets [19] and the primary mode of transmission of SARS-CoV-2 are outside the scope of this study. The size ranges for aerosols and droplets were adopted based on the instruments used for characterization.

## Materials and methods

### Choice of fabrics

Materials were chosen to represent the breadth of the materials covered in the literature (Fig A in S1 Text). Very high thread-count cotton materials (1000 threads per inch, or TPI) that have received the least attention were added to the list as well. The list of those materials, including their composition, areal density and thickness are reported in Table 1. Areal density of the fabrics was measured by cutting small coupons (5 cm x 5 cm) and then weighing them on a balance (Scientech SA 310 –Boulder, CO). The thickness measurements of the fabrics were performed using a digital indicator with a 25 mm diameter contact plate (Mitutoyo 543–256 –Aurora, IL). Except for the cellulose-based absorbent materials and the recyclable handbags, all other fabrics were either woven (all bedsheets, pillowcases) or knit (cooling scarf, scarf, mask bandana, neck tube, T-shirt, washcloth) (Fig B in S1 Text).

**Table 1:** Different materials selected, and their properties including dry filtration efficiencies, and pressure drop at velocity 9 cm/s.

Description (Alias)	Composition	Thickness (mm)	Areal Density (g/m2)	Filtration Efficiency (%)	ΔP in mmH_2_O (Pa)
N95	-	-	-	98.84 ± 0.49	5.8 ± 0.8 (57)
One Thousand TPI Bedsheet—2 (1000 TCBS2)	100% cotton	0.389 ± 0.011	191.3 ± 2.3	53.34	32 (314)
One Thousand TPI Bedsheet—1 (1000 TCBS1)	100% cotton	0.277 ± 0.008	150.6 ± 2.9	48.95 ± 1.16	27.7 ± 3.2 (272)
One Thousand TPI Pillowcase (1000 TCPC)	100% cotton	0.317 ± 0.005	182.6 ± 0.9	41.62 ± 3.2	23.6 ± 1.5 (231)
Blue Jeans	100% cotton	0.970 ± 0.005	400.9 ± 5.2	40.52 ± 3.61	20.1 ± 2.3 (197)
Microfiber pillowcase—1 (Microfiber PC1)	100% polyester	0.167 ±0.003	81.6 ± 0.7	30.82 ± 6.58	20 ± 1.5 (196)
Canvas dropcloth	Cotton blend	0.778 ± 0.015	338.9 ± 6.9	18.89 ± 3.77	5.9 ± 0.6 (58)
Recyclable Handbag	100% Polypropylene	0.510 ± 0.008	82.9 ± 0.1	14.08 ± 1.14	0.9 ± 0.0 (9)
Silk Pillowcase	100% mulberry silk	0.180 ± 0.002	80.1 ± 1.7	12.90 ± 12.99	1.1 ± 0.1 (11)
Two hundred TPI Pillowcase	60% cotton, 40% polyester	0.307 ±0.009	117 ± 0.5	9.94 ± 3.75	1.1 ± 0.2 (11)
Six hundred TPI Bedsheet	100% cotton	0.281 ± 0.008	128.9 ± 0.4	8.70 ± 1.50	1.9 ± 0.2 (19)
Wash cloth	87% cotton, 13% polyester	2.203 ± 0.114	320 ± 12.3	7.89 ± 1.66	0.5 ± 0.0 (5)
Flannel Bedsheets	100% cotton	0.706 ± 0.003	176.9 ± 0.9	7.32 ± 4.56	1.1 ± 0.0 (11)
Microfiber Pillowcase—2	100% polyester	0.285 ± 0.007	98.9 ± 1.8	7.12 ± 4.57	2.1 ± 0.0 (21)
Neck tube	92% polyester, 8% spandex	0.536 ± 0.003	132.2 ± 2.1	7.10 ± 3.92	1.4 ± 0.2 (14)
Polypropylene	100% Polypropylene	0.363 ± 0.008	51.4 ± 1.6	6.58 ± 1.58	0.5 ± 0.0 (5)
Face tissue paper	100% cellulose	0.171 ± 0.004	31 ± 0.4	4.57	2.0 ± 0.0 (20)
Scarf	63% Acrylic, 30% nylon, 7% wool	5.140 ± 0.057	449 ± 9	3.79 ± 2.27	0.5 ± 0.1 (5)
T-shirt	100% cotton	0.523 ± 0.007	144.1 ± 2.9	3.68 ± 2.79	0.6 ± 0.2 (6)
Paper towel	100% cellulose	0.650 ± 0.020	57.9 ± 0.5	3.34	1.2 ± 0.1 (12)
Cooling scarf	100% polyester	0.645 ± 0.010	141.1 ± 2.8	2.94 ± 1.28	0.2 ± 0.0 (2)
Mask bandana	100% microfiber polyester	0.311 ±0.006	142.1 ± 3.1	1.52 ± 0.44	0.2 ± 0.1 (2)

### Step 1: Filtration efficiency of fabrics against submicron dried sodium chloride aerosols

**Fig 1:**
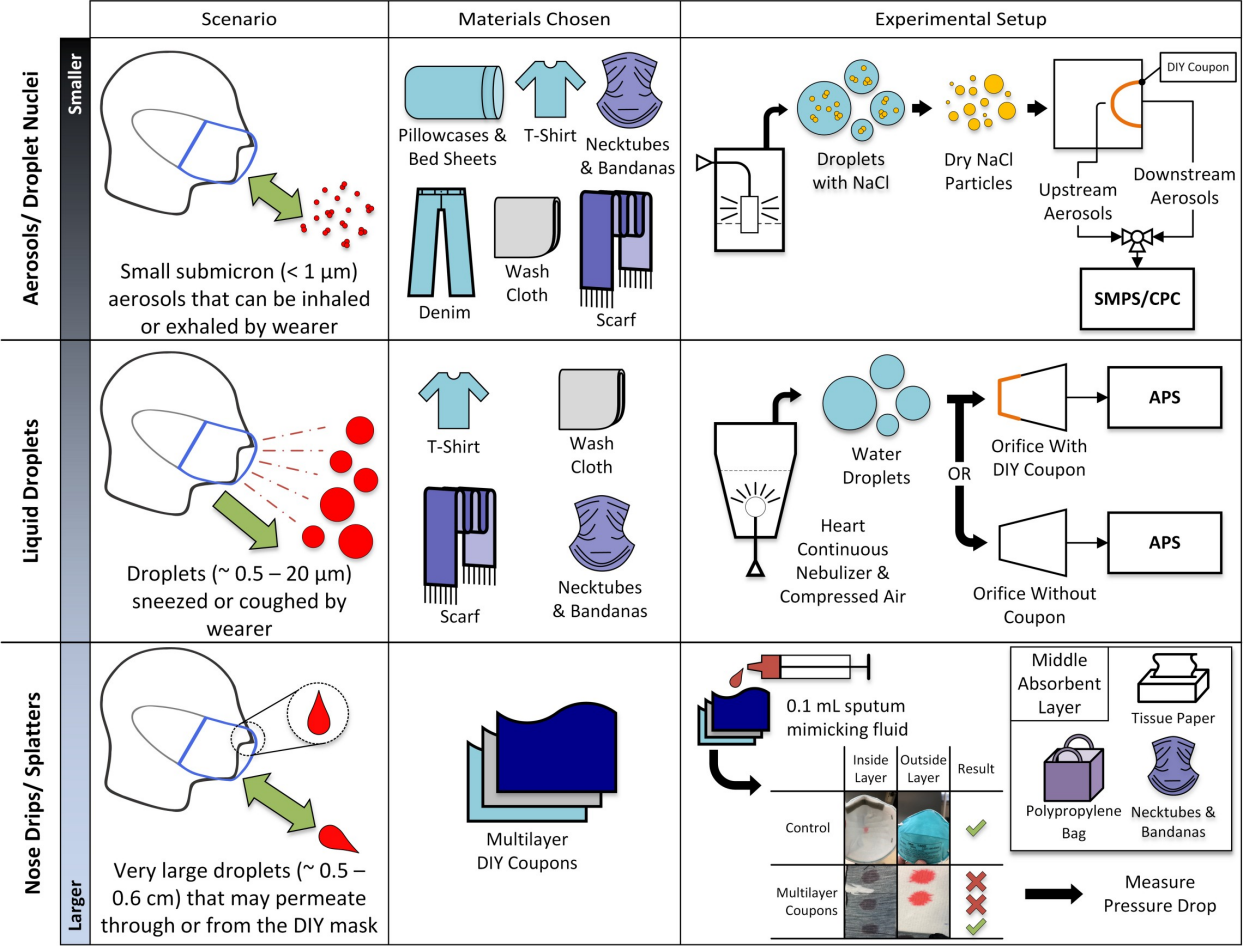
An overview of the three-step methodology. *Step 1*: Household material coupons were tested for filtration efficiency and pressure drop at a flow velocity of 9 cm/s using dried, charge conditioned aerosols (Table 1). *Step 2*: Select materials were tested against wet water droplets at very high velocities (~ 481 cm/s and 1700 cm/s) (Fig 2). *Step 3*: a large volume (0.1 mL) of artificial saliva was deposited on the material surface to investigate the permeability of multilayered DIY coupons (Table 2). SMPS: Scanning Mobility Particle Sizer; CPC: Condensation Particle Counter; APS: Aerodynamic Particle Sizer; DIY coupons: Do-It-Yourself i.e. household material coupons.

Fig 1 shows the three-step approach used for performance testing of the household materials. The coupons were taped and sealed onto a flat plate using adhesives. The coupon sizes were cut such that the face velocity through the fabrics would be approximately 9 cm/s, which would match the velocity typically used when certifying N95 respirators. The schematics of the experimental set up, sampling biases, and time to attain steady state concentration are provided in Figs D through F in S1 Text. The relative humidity in the chamber that housed the fabrics was maintained at 18 ± 4%. To mitigate the intra-material variability, coupons were cut from different batches. The filtration efficiency (FE) experiments were performed predominantly in triplicates except for 1000TCBS2, face tissue and paper towel. The FE experiment with 1000TCBS2 was abandoned after an excessively large pressure drop was measured. The primary purpose of the cellulose materials was for absorption of fluids; therefore, they were also not studied in triplicates after they were identified to have low dry FE (< 10%). However, pressure drop for the cellulose based materials were made in duplicates.

Polydisperse sodium chloride (NaCl) aerosols were generated by nebulizing 1% NaCl, then dried (TSI Inc., Model # 3062, Shoreview MN) and charge reduced (TSI Inc., Model # 3054 Shoreview MN) before sending to a large, well-mixed chamber. Consistent with prior studies [38,39], and NIOSH 42 Code of Federal Regulations 84 used for certifying N95 respirators, [40] this step generated aerosols approximately 80–90 nm in diameter (Fig G in S1 Text). The flow rates were maintained using a mass flow controller (Alicat Instruments Model 64865). A Scanning Mobility Particle Sizer (SMPS, TSI Inc., Model # 3936) (Fig C in S1 Text) was used at sheath flow rate of 3.0 L/minute, to sample upstream and downstream of the material coupons at a rate of 0.3 L/minute in the mobility size range of ~ 15–661 nm after the chamber had reached steady state. The sampling bias within this size range remained typically within ± 5% (Fig F in S1 Text). The SMPS was calibrated with 101 and 505 nm National Institute of Standards and Technology (NIST) traceable monodispersed polystyrene latex beads (Polysciences Inc.).

The scan size ranged from 15.1 nm, which is lower than the smallest size of SARS-CoV-2 [41], to 661 nm. The scanning time for one complete size distribution with the SMPS was roughly 135 seconds, with 120 seconds of up scan (when the voltage in the DMA would increase) and 15 seconds of down scan (when the voltage in the DMA would quickly decrease to near zero). Scans were first run for three consecutive times for the downstream probe followed by the upstream probes. Validation experiments were performed with one N95 respirator model (Fig H in S1 Text). The filtration efficiency for a fabric would be determined by using equations presented later.

Differential pressure was recorded at 8 Hz using a custom data acquisition (DAQ) system with 16-bit analog to-digital converters and unidirectional low-range differential pressure transducers. Two different ranges of pressure transducers were utilized (OMEGA, Norwalk, CT); 2.0 inH_2_O (498 Pa) (PX165-002U) and 0.25 inH_2_O (62 Pa) (PX165-0.25U). The accuracy for the pressure transducers is provided as ±1% full scale. Sample time and pressure (mmH_2_O) were recorded from the DAQ using a Python data logging interface and saved to a text file.

For all three tightly woven cotton fabric brands, such as the one thousand TPI pillowcases and bedsheets, the denim jeans, and Microfiber PC1, it was observed that the 1% NaCl would saturate the fabrics in approximately 5–10 minutes. The associated pressure drop would rapidly exceed the maximum pressure range of ~50 mmH_2_O (inset of Fig J in S1 Text). Because the SMPS needs a steady-state size distribution and its acquisition rate was too slow to capture this fabric saturation phenomenon, for the tightly woven fabrics the SMPS was replaced with the particle counter (TSI Model 3775 Shoreview MN), which has an acquisition rate of one data point per second. The counter enabled monitoring of the concentration downstream of the fabric as a function of time until the pressure drop started to increase. The concentration downstream of the fabric would be measured for approximately 1 minute (post steady state) and then the valves were switched upstream to measure the concentration upstream of the fabric. To reduce the artificial inflation of pressure, drop and filtration efficiency for such fabrics, only the initial values of filtration efficiency and pressure drop are reported. Standard deviations are calculated from measurements made predominantly in triplicates, unless mentioned otherwise.

### Step 2: Blocking efficiency of fabrics against micro-droplets during sneezing

The velocity of droplets generated by talking, coughing, and sneezing can vary widely with subjects, with different types of activities. These droplets are much larger in size compared to sub-micron aerosols [33]. To characterize these larger droplets, an Aerodynamic Particle Sizer (APS, TSI Inc, Model 3321 Shoreview, MN) was used. Sneeze velocities can range from hundreds of cm/s to few thousand cm/s [33]. Two values in this range were chosen: 481 cm/s (4.81 m/s) and 1700 cm/s (17.00 m/s). For a fixed suction flow rate of 5 L/minute used by the APS, these velocities were achieved by using orifices of 0.47 cm and 0.25 cm diameter, respectively. A schematic of the test setup is shown in Fig M in S1 Text. The droplet (also referred to as wet or blocking) FE of fabrics was determined using water droplets with mean aerodynamic diameter of 3.4 to 3.9 μm. generated by a Heart Continuous Nebulizer (Westmed Inc. Tucson AZ). With a quick scan rate of 15 s, the APS enabled monitoring of the wet FE dynamically–from when the fabric was completely dry (at the beginning of these experiments), partially wet (around 1 minute later), and later completely wet (about 2 minutes later).

The large velocities also resulted in significant pressure drop across the fabrics (Fig P in S1 Text). In order to ensure that these pressure drops did not impact the sizing accuracy of the APS, it was calibrated with NIST traceable 3.0 μm polystyrene latex beads (PSL) over the entire pressure drop range exhibited by the fabrics investigated (Fig O in S1 Text).

Experiments were performed either by mounting the fabric onto the orifice in line with the APS suction flow, or without the fabric. At least four trials (n≥4) with four coupons were conducted for each fabric type. To limit the total number of experiments, low-pressure-drop fabrics were primarily chosen for these experiments, as they were likely to fare worse than the fabrics that had higher pressure drops and higher dry FE. Only one tightly woven fabric, a one thousand TPI pillowcase was used. One medical grade facemask cleared by US Food and Drug Administration (FDA) was used as a control.

The droplet size distribution was measured by the APS approximately 0.74 meters downstream of the location of the fabric. To rule out any sizing biases because of significant drying of the droplets during transit from the fabric holder to the APS, an additional set of droplet size measurements were made with a much shorter (0.1 meters) tubing length as well (Fig N in S1 Text).

### Step 3: Permeability test of fabrics against macro-droplets such as large splatters and nose drops

In the final step, the permeability of the DIY materials was tested with commercially available artificial saliva [42] (Biotene dry mouth oral wash, GlaxoSmithKline–Brentford, UK) on the multilayered DIY coupons. To determine permeability, a 0.1 mL droplet of artificial saliva was placed on the first of several layers of material stacked vertically to allow gravity to aid the permeation. The number of material layers was increased until the droplet was unable to permeate the stack of layers. A pass-fail criterion was assigned using a fluid volume of 0.1 mL, the amount of sputum expected to be generated by a person coughing over a span of 1 hour. To aid visualization, the artificial saliva was dyed red. The permeability of various materials was monitored for over 1 hour, at the end of which the layers were separated while visual inspection for permeation was performed. The pressure drop of the multilayered materials was measured using the same test set up that was used for single layered fabrics and is already described in step 1 above. The fabrics in this step typically did not undergo sub-micron fabric performance testing using NaCl.

### Data analysis

For step 1, the size-based penetration % of the materials was obtained using:

$$Penetration(d)=\frac{C_{downstream}(d_{mobility})}{C_{upstream}(d_{mobility})}\times 100\%$$ (1)

where C_downstream_ and C_upstream_ are the concentrations sampled by the downstream and upstream probes, at a specific bin size d_mobility_. The penetration is plotted for some household materials in Fig H in S1 Text. Standard deviations are from measurements made in triplicates. The average filtration efficiency (FE_avg_) (%) of the materials was then determined from:

$${FE}_{avg}=1-\frac{1}{N}\sum_{i\geq 0.015\;\mu m}^{i<0.661\;\mu m}Penetration\;(d_{size})$$ (2)

where N is the corresponding number of bins and was equal to 100 and is reported in Table 1. For the tightly woven fabrics, a single penetration efficiency was obtained for the entire range of sizes (since a particle counter cannot delineate size), and thus it did not require any averaging.

For droplet filtration efficiency measured only with the APS for large droplets, the APS was scanned every 15 seconds for up to 1 minute without fabrics, and 2 minutes with fabrics. The additional 1 minute with fabrics was used to determine if the continued wet state of the fabrics impacted the droplet FE. After mounting the coupon, the first scan was not considered, to allow time for the droplets to reach the APS. For the scanning performed from 16–30 s, the equation for penetration is given as follows:

$$P(d_{ae},15\;s)=\left(\frac{C_{with\;fabric}(d_{ae},t)}{\frac{1}{3}\sum_{i=15\;s}^{i=60\;s}C_{without\;fabric}(d_{ae},t)}\right)\times 100\%$$ (3)

where d_ae_ is the aerodynamic diameter. The averaged values are divided by three to represent the three 15 second runs at 16–30 s, 31–45 s and 46–60 s. Standard deviations are for measurements performed with n≥3.

**Fig 2:**
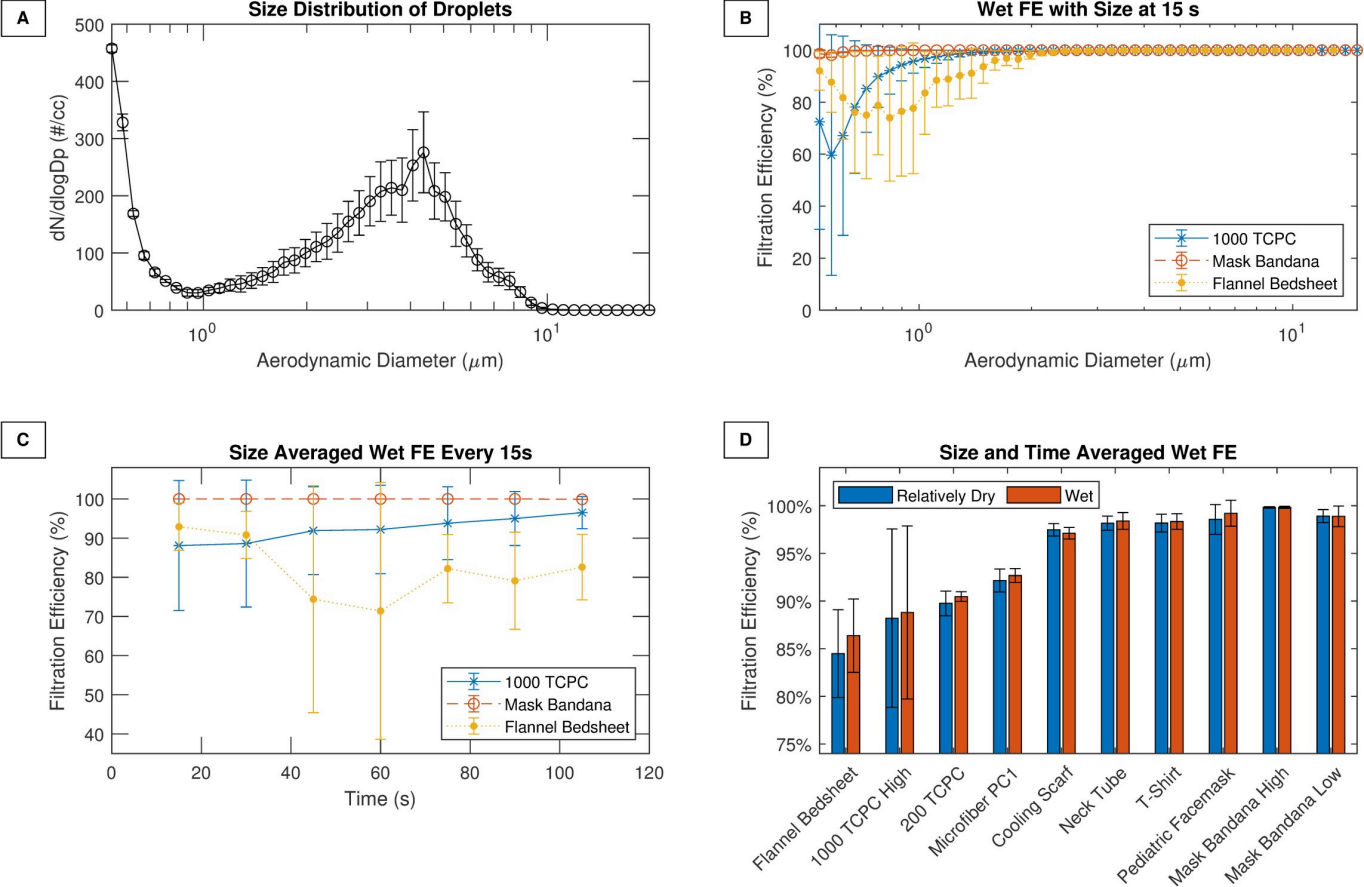
Fabric performance against droplets. (A) Size distribution of the droplets generated for wet FE experiments. (B) FE as a function of size for three fabrics during first 15–30 seconds. (C) The size-based FE averaged over the entire size range and plotted for each scan interval from 15 s to 120 s for the same materials. (D) The time-averaged and size-averaged droplet filtration efficiency for various materials. A medical grade facemask was also used as a control. TC: Thread count; PC: Pillow case. High–represents high velocity (~ 1700 cm/s).

The size-averaged droplet FE, say at 15 seconds, obtained with the APS, is given by:

$${FE}_{avg}(15\;s)=\frac{1}{N}\sum_{i=0.542\;\mu m}^{i=10.37\;\mu m}\left(1-\frac{C_{with\;fabric}(d_{ae},15\;s)}{C_{without\;fabric}(d_{ae},15\;s)}\right)\times 100\%$$ (4)

where N is the number of bins in the size range of 0.542–10.37 μm and equaled 41 bins. Bins beyond 10.37 μm were not considered as the concentration for such bins were almost often too low to extract any statistically meaningful filtration efficiency. The total FE was then integrated from 15 s– 60 s (fabric relatively dry) and then from 60 s– 120 s (fabric wet) duration to obtain the overall FE_avg_ of the fabric at 481 cm/s or 1700 cm/s and is reported in Fig 2D. An example time-averaged and size-averaged FE equation for relatively dry fabric is:

$${FE}_{avg-fabric\;relatively\;dry}=\frac{1}{N}\sum_{j=0.542\;\mu m}^{j=10\;\mu m}(1-\frac{\frac{1}{3}\sum_{i=15\;s}^{i=60\;s}C_{with\;fabric}(d_{ae},t)}{\frac{1}{3}\sum_{i=15\;s}^{i=60\;s}C_{without\;fabric}(d_{ae},t)})\times 100\%$$ (5)

## Results and discussion

### Step 1: Performance of household materials against submicron aerosols

The average FE’s of sub-micron, solid, charge-conditioned, sodium chloride aerosols, henceforth referred to as dry FE, and the pressure drop (ΔP in mmH_2_O with error bars and Pa without error bars) are reported in Table 1. Twenty-one single-layer materials are featured in this table, along with a N95 respirator used as a control. Only five fabrics achieved decent dry FE exceeding 30% at sub-micron size. Such materials were tightly woven (Fig B in S1 Text) and typically had high reported TPI if they were made from cotton. Multiple 1000 TPI cotton brands were investigated to determine the impact of brand variability. The differences across these brands were minimal (Table 1) with most offering FE of 40–50%, comparable to that of low filtration surgical masks [22]. Interbrand variability for the tightly woven high TPI cotton coupons was likely due to differences in manufacturer reported TPI, unknown coatings or treatment of materials. Overall, the fabrics showed good agreement with data reported by others (Fig L in S1 Text).

Prior studies with different materials showed significant variability in dry FE (Fig A in S1 Text). The values reported here are in good agreement with literature (Fig K in S1 Text) [5,6,22,43]. Also consistent with prior NIOSH investigation, and a more recent NIST study [5,22], we found a size-dependency for filtration for several fabrics (Fig H in S1 Text) with maximum penetrating particle size exceeding 200 nm. This size dependency is also seen in mechanical filters. To confirm if the fabrics capture particles only by mechanical means, or if electrostatics also plays a role, we subjected 1000TCPC to isopropanol treatment (IPA). The presence of any electret filters or electrostatic charges would have shown a clear reduction in dry FE [32]. However, the FE of the non-IPA-treated versus IPA treated samples were not statistically different (student t-test, p value = 0.17, Fig I in S1 Text), indicating that mechanical filtration is the likely mechanism of capture by the tightly woven cotton fabrics.

### Step 2: Performance of fabrics against droplets at high velocities

For characterizing the filtration efficiency of larger droplets (> 0.5 μm) at high velocities, aerosols in the size range of 0.5–20 μm were generated. This range has been reported (Fig 2A) to be the range for droplets generated by coughing [44,45]. The FE versus size plot in Fig 2B is shown for three fabrics at 16–30 s after the start of the experiment, when the fabrics are relatively dry. The mask bandana and 1000 TCPC were tested at a velocity of 1700 cm/s, and the cotton flannel was tested at 481 cm/s. The 1000 TCPC and cotton flannel showed the characteristic U-shaped efficiency versus size curve that is expected of filters. But the mask bandana had wet FE that exceeded 99% across all sizes. Its hydrophobic properties (contact angle exceeded 90 degrees, Fig R in S1 Text) may explain why it was able to efficiently capture the droplets. Fig 2C shows the size-averaged wet FE as a function of time for the same three materials. While the cotton flannel wet FE drops somewhat during intermediate time points, it recovers again. For the other materials the wet FE remains virtually the same during the entire time. Thus, it can be inferred that the wet efficiency does not seem to change significantly over time as the material transitions from being dry (15 seconds), to partially wet (~ 1 minute) to wet (~ 2 minutes). This implies that fabric materials may be able to continue to offer protection over multiple events of sneezes or coughs, and the humidity from exhaled breath may not significantly impact droplet FE.

Fig 2D reports the time-averaged and size-averaged droplet filtration efficiency of several materials that fared poorly with dried and sub-micron aerosols. Several materials were found to have > 90% droplet filtration efficiency, which is comparable to medical-grade masks. Wet FE data for the first minute for several fabrics is compared against published values in supporting information (Fig Q in S1 Text). Good agreement was observed with recent data collected using completely different techniques, including visualization and fluorescence [30,46]. It can be inferred that at very high, sneezing-like velocities, even single layers of materials (e.g. T-shirt, bandanas, cooling scarfs) that have poor FE against sub-micron aerosols can offer significant protection against larger (> 1.0 μm) wet droplets, and may be effective source controls.

### Step 3: Performance of fabrics against macro droplets, large splatters or drops

To identify materials that would pass the three criteria of high dry FE, high droplet FE, and the permeability, three options were considered: multiple layers of the same materials, combination materials with fabrics, and combination materials with fabrics and an intermediate highly absorbent layer made up of cellulose type materials. Table 2 reports the pass-fail results from several of the permeability tests, with additional results provided in supporting information (Table A of S1 Text). Pictures from an example study are provided in Fig S in S1 Text. As expected, N95s, used as controls, passed such tests. Most single layered materials, independent of their dry FE, failed. An exception was polypropylene (Table A in S1 Text). In some cases, even triple layered materials failed the permeability test (Table 2). This underscores the importance of using at least three or four layers of household materials when making face coverings. The mask bandana did well, likely because of its hydrophobic properties. The advantage of this specific fabric material was that adding multiple layers does not significantly increase the pressure drop (Table 1). Hence this material was further explored for creating combination materials. Absorbents (e.g. tissues, towels, toilet paper) as a middle layer may be effective for reducing permeation of supra large droplets such as splatters, nose drips etc. (Table 2), despite having low dry FE (Table 1).

**Table 2:** Layers of materials, or combination materials, from wearer side to the outside, rate of passing the permeability test, and the corresponding pressure drop.

Inner Layer–Middle Layer(s)—Outer Layer	Permeability Test (Pass/Total Tests)	Pressure Drop in mmH2O (Pa)
1000 TCPC—1000 TCPC—1000 TCPC—1000 TCPC	3/3	-
Microfiber PC1—Microfiber PC1—Microfiber PC1—Microfiber PC1	3/3	-
1000 TCPC—1000 TCPC—1000 TCPC	0/3	> 50.8 (498)
Microfiber PC1—Microfiber PC1—Microfiber PC1	0/3	> 50.8 (498)
1000 TCBS1—Mask Bandana—Mask Bandana—Mask Bandana	3/3	29.6 ± 1.0 (290)
1000 TCPC—Toilet Paper—Mask Bandana	3/3	29.5 ± 1.6 (289)
1000 TCBS1—Mask Bandana—Mask Bandana	3/3	28.7 ± 1.2 (282)
1000 TCPC—Tissue Paper—Mask Bandana	3/3	29.5 ± 0.4 (289)
1000 TCPC—Mask Bandana—Mask Bandana	3/3	27.0 ± 0.5 (265)
Mask Bandana—Mask Bandana—1000 TCPC	3/3	-
MicrofiberPC1—Mask Bandana—Mask Bandana—Mask Bandana	3/3	24.6 ± 0.8 (241)
MicrofiberPC1—No Middle Layer—Mask Bandana	5/6	19.6 ± 0.3 (192)
1000 TCPC—Paper Towel—Mask Bandana	3/3	30.6 ± 0.4 (300)
1000 TCPC—Mask Bandana—Mask Bandana—Mask Bandana	3/3	27.4 ± 0.6 (269)
Mask Bandana—Mask Bandana—Mask Bandana—1000 TCPC	3/3	-
Mask Bandana—Mask Bandana—Microfiber PC1	2/3	-
MicrofiberPC1—Mask Bandana—Mask Bandana	3/3	22.0 ± 1.6 (216)
Mask Bandana—No Middle Layer—Microfiber PC1	0/3	-
N95 Respirator	3/3	5.8 ± 0.8 (57)
200 TCPC- 200 TCPC—200 TCPC—200 TCPC	3/3	4.1 ± 0.2 (40)
2000 TCPC- 200 TCPC—200 TCPC	2/3	2.8 ± 0.2 (27)
T-shirt—T-shirt—T-shirt—T-shirt	3/3	2.7 ± 0.3 (26)
Pediatric Facemasks	3/3	2.1 ± 0.2 (21)
T-shirt—T-shirt—T-shirt	3/3	1.8 ± 0.0 (18)
Mask Bandana—Mask Bandana—Mask Bandana—Mask Bandana	3/3	1.2 ± 0.1 (12)
Mask Bandana—Mask Bandana—Mask Bandana	3/3	0.7 ± 0.1 (7)
Cooling Scarf—Cooling Scarf—Cooling Scarf—Cooling Scarf	3/3	0.7 ± 0.1 (7)
Cooling Scarf—Cooling Scarf—Cooling Scarf	0/3	0.4 ± 0.0 (4)

### Breathability

Because breathability is a highly subjective metric, clinical studies would be valuable in evaluating the ease to which breathing can be performed. In absence of any such comprehensive studies, we can interpret the breathability of the fabrics we tested by comparing with N95s, surgical masks and pediatric facemasks. The maximum permissible inhalation resistance of N95 respirators is 35 mmH_2_O for adults [47]. For children, prior bench top studies have determined that during light activities, the pressure drop across children’s facemasks, including those cleared by the FDA, ranges from 1.2–1.8 mmH_2_O at 30 L/minutes. This is similar to the pressure drop for medical-grade surgical masks [38,48]. Based on Table 1, a clear trend emerges–the fabrics studied can be categorized in to two groups: highly breathable, loosely knit or woven fabrics that have pressure drop comparable to pediatric facemasks (21 Pa), and less breathable, tightly woven fabrics, with pressure drops comparable to the limit of N95 respirators (343 Pa). Given that none of the single layers of loosely or tightly knit/woven fabrics would pass the permeability test (Table A in S1 Text), multiple layers would be desirable. The pressure drop for such multilayered materials is provided in Table 2. For tightly woven fabrics, the increase in the pressure drop with increasing number of layers is evident, exceeding the maximum measurable limit of the pressure gauges in several instances.

Since choosing even a single layer of tightly woven fabrics (Table 1) for children would mean the inhalation-resistance offered by these fabrics would be ten-fold more than pediatric facemasks, caution should be exercised when making such choices. Without tightly woven fabrics, options for protecting the pediatric wearer would be limited, as the loosely knit or low thread count fabrics rarely offer dry FE > 10%. For source control, three to four layers of loosely knit or woven cotton, or polyester fabrics, would be acceptable choices. Even with multiple layers, their pressure drop is comparable to pediatric facemasks (Table 2). Such multilayered, highly breathable fabrics will also protect wearers and neighbors against macro-droplets (e.g. from fomites) (Table 2).

Adults have more choices. Assuming pressure drop linearly increases with number of layers [5], for many of the tightly woven fabrics two or more layers (Table 2), or in some cases even single layers (1000 TCBS2) (Table 1), may equal the 35 mmH_2_O inhalation resistance limit. This implies, for adults, when choosing multiple layers of tightly woven fabrics, caution should be exercised. An alternative is a combination of one tightly woven fabric layer with other, easier-to-breathe layers of cotton, polyester, nylon or blends. As seen from Table 2, multiple material combinations can be used by adults without exceeding the 35 mmH_2_O limit. One inner layer (i.e. on the wearer’s side) of 1000 TPI cotton, with two to three layers of hydrophobic polyester, or one inner layer of 1000 TPI cotton, combined with one intermediate layer of absorbent cellulose material and one-layer hydrophobic polyester, (or similar combinations with high filtration microfiber replacing 1000 TPI cotton) may be tolerable for adults. Given the relatively decent FE (~ 40%) of the 1000 TPI tightly woven fabrics, some protection to the wearer may be expected in such cases.

For choosing hydrophobic or absorbent middle layers, it is noted that cellulose materials such as tissue papers, toilet papers paper towels, and polypropylene cutouts from recyclable bags would not significantly add to the pressure drop (Table 1). Whether high velocity from sneezing will tear and compromise the paper layers was beyond the scope of this study.

### Reusing face coverings

Currently there is a dearth of information on how fabrics may fare when cleaned before reuse. Recent WHO guidance [3] suggests that cloth fabrics may be boiled or steamed. In order to characterize the impact of boiling on the dry FE of a combination material that passed all three tests 1000 TCPC– 3 layers of mask bandana, this combination material was boiled and retested. The findings suggest that a 10 or 60-minute boiling does not impact the FE of these materials (one-way ANOVA p = 0.507, Fig T in S1 Text). The permeability of the 1000 TPI cotton with three layers of mask bandana was further characterized; its permeability to macro droplets remain unchanged after boiling (Table A in S1 Text). Thus, combination materials such as 1000 TPI cotton-mask bandana can be re-boiled at least up to 60 times. This is assuming 1-minute boiling is enough for inactivating the accumulated bioburden in a face covering from a single use and that repeated heating and cooling cycles do not fatigue the fabric fibers. If choosing paper-based materials as middle layers, given their lower strength, it would be best to introduce a new sample before each reuse.

Information on performance of face coverings with repeated reuse is sparse. Therefore, while our results did not show difference in filtration efficiency (Fig T in S1 Text) and permeability (Table A in S1 Text) from boiling, general inferences should be made with caution. Note that donning and doffing of face-coverings, prolonged exposure to humidity, sneezing or coughing or chemical decontamination methods may potentially impact fabric fibers or the strap integrity. Clinical studies comparing subjects wearing fresh face coverings and decontaminated face coverings made from the same fabrics may provide insights into the real-world performance of face coverings with repeated reuse.

### Study limitations

Experiments were performed before washing of the newly acquired fabrics, and washing may have some impact on the reported findings. Constant flow rates were used for dry and wet FE experiments, which is not clinically representative of the sinusoidal breathing patterns, nor of the instantaneous nature of sneezing. The maximum size range of the SMPS used was less than 0.7 μm, and thus the FE for the dry aerosols beyond that range are not reported here. Given that the maximum penetrating particle size, and the minimum FE for fabrics, typically occurs below 0.7 μm size, [5] and subsequently FE increases with increase in particle size above 0.5 μm (Fig A in S1 Text), the fabrics reported here are likely to fare better at the larger sizes, offering more protection to the wearer.

FE’s under 0.542 μm were not studied, as the APS cannot characterize aerosols or droplets below that size. Talking or mild activities are likely to generate such droplets. While the smaller size may reduce filtration efficiency of such droplets, the lower velocities, and the charges on these droplets may compensate and enhance the capture. More studies for droplets under 0.50 μm may be needed to confirm this hypothesis.

Although 35 mmH_2_O is a well-accepted pressure threshold and it was used for selecting combinations of materials, even this limit has been associated with complaints of discomfort and headaches [49–51]. In order to determine how low the pressure drop for N95s need to be, CDC performed a number of clinical studies and concluded that the pressure drop needs to be < 9 mmH_2_O (88.2 Pa) to ensure that the N95s do not pose breathability issues even during moderate activities [50]. The findings of this CDC study were applicable only to adults. It is also noteworthy that this pressure drop threshold is consistent with the latest WHO guidance on non-medical masks, which stipulates that the pressure drop be < 100 Pa [52]. None of the tightly woven fabrics characterized here meets that criterion. For children, a lower pressure drop is desirable. In order to gauge the tolerance of tightly woven fabrics such as 1000 TPI pillowcases and bedsheets, it would be useful to conduct further studies with human subjects.

The impact of the fit with the face of different mask designs, and the effect of strap tensions, on total inward leakage of aerosols was not investigated. The total inward leakage would be strongly dictated by the breathability of the fabric, with more leaks for less breathable materials [38]. In a real-world scenario involving a face covering, sneezing may create a large pressure drop, even up to 3000 mmH_2_O (Fig P in S1 Text) for a brief period, which may cause temporary leaks at the face and face covering interface. Our lab-based study did not consider this scenario. Future studies with orthogonal visualization techniques [53] may be able to quantify this leakage.

For the wet FE experiments, droplets that were not dried or charge conditioned were intentionally used, and these droplets may have possessed excessive number of charges [54]. Polarization of the fabric fibers may have occurred while the droplets passed through the coupons, and some particle capture by electrostatic attraction may have occurred. Because it is not known how the charges in water droplets compare with charges in mucous droplets generated during coughing or sneezing, further investigation in this area is desirable. Droplets harboring microorganisms have been reported to have thousands of charges [55], and it is possible that even clinically, charges may enhance capture of the cough or sneeze droplets on fabrics.

While re-aerosolization from fabrics was outside the scope of this study fragmenting of droplets have been reported elsewhere and warrants further studies [56]. In addition, while aerosols generated by singing and talking have received recent attention [16], other actions such as yawning, smoking etc. can also potentially generate aerosols and droplets. Consstribution from such actions towards airborne transmission was not covered in this study.

## Conclusions

Face coverings, 3D printed facemasks, and surgical masks play a significant role in combatting infection during emergency situations, when N95 respirators are not available. Of these, face coverings made from household fabrics are likely to be most easily available. Since the beginning of the coronavirus pandemic, several studies have characterized the filtration properties of household fabrics, either in context of aerosols or droplets. Few studies have characterized fabrics across all the major modes of transmissions. In addition, while cotton is a popular choice of fabric, very few studies have characterized the performance of high TPI cotton. The present investigation utilized a comprehensive fabric-characterization methodology that accounts for three major modes of transmission of airborne respiratory viruses such as SARS-CoV-2. Testing was conducted with various single-layer and multilayered fabrics in the size range of 0.02–10 μm. The major findings of the study can be summarized as follows–

- Loosely knit or woven household fabrics made of cotton, polyester, nylon, spandex or blends:
  - Even with multiple layers, would be as breathable as medical grade masks such as pediatric facemasks, and would protect wearer against macro-droplets.
  - Because of low dry filtration efficiency, these materials will offer very little protection to wearer.
  - May offer good protection against droplets even at high velocities, and act as effective source controls.
  - May be best option for pediatric population.
- Tightly woven, one thousand thread count household fabrics made of cotton:
  - Even single layer would have a breathing resistance comparable to N95 respirators, and therefore multiple layers of such fabrics should be avoided. Instead, such materials can be combined with other loosely knit materials.
  - Filtration efficiencies for sub-micron aerosols can exceed 40%, indicating that the coverings offer some level of protection to the wearer.
  - Can offer protection against micro-droplets during sneezing and coughing.
  - When combined with other loosely knit and highly breathable materials, can offer protection against macro-droplets.
  - Combination materials made from such fabrics retain their dry filtration efficiency and macro droplet blocking abilities even after sixty minutes of boiling.
  - Such material combinations are almost ten times harder to breathe through compared to medical grade pediatric masks, and thus would be best to avoid for children.

## Supporting information

S1 Text Supporting information for comprehensive characterization of protective face coverings made from household fabrics. (PDF)

S1 Data Supporting information containing data. (XLSX)
