# Supplementary material for: Comprehensive characterization of protective face coverings made from household fabrics
Source: PLoS One. 2021 Jan 13;16(1):e0244626. doi: 10.1371/journal.pone.0244626 (PMC7806137; doi:10.1371/journal.pone.0244626)
Supplement: S1 Text — (PDF) [file pone.0244626.s001.pdf]

Supporting Information

for

Comprehensive Characterization of Protective Face Coverings Made from Household Fabrics

Suvajyoti Guha[1]*, Alexander Herman[1], Ian A. Carr[1], Daniel Porter[1], Rucha Natu[1], Shayna Berman[1], Matthew R. Myers[1]

[1]Division of Applied Mechanics, Office of Science and Engineering Laboratories, Center for Devices and Radiological Health, United States Food and Drug Administration, 10903 New Hampshire Avenue, Silver Spring, MD 20993.

*Corresponding Author

Email: Suvajyoti.Guha@fda.hhs.gov

Short Title: Comprehensive characterization of Fabrics for Use as Face Coverings

Material Selection and Micrographs

Household Material Selection Criterion

The keywords: “homemade face masks” “cloth masks” “face masks” “respiratory protection” “personal protection” “SARS-CoV-2” “COVID-19” “Coronavirus” “inhalation exposure” were used in the Google search engine and in Google Scholar. Several peer-reviewed studies and non-peer reviewed literature on filtering efficiency of common materials found in the home were included.[1-10] Non-peer reviewed and peer-reviewed studies focused fit, leakage, or other confounding factors of mask effectiveness, apart from the filter properties of materials, were excluded. Figs A and B summarize the literature and categorize the fabrics tested. The key takeaways were:

a) Densely woven materials tend to have higher filtrations efficiency than looser weaves.
b) No single category of fabric necessarily performs better than others. Filtration efficiency appears to depend on the specific characteristics/manufacture of the fabric, i.e. weave density rather than simply the fiber material.
c) Droplet filtration efficiency tends to be significantly higher compared to the dry filtration efficiency.

Further research was conducted to evaluate how these household materials are being used to make face coverings. DIY masks recommended by healthcare providers and children’s hospitals were included. [11-18] The key takeaways were -

a) Cotton fabric (100% cotton or polyester cotton blends) is recommended for DIY masks in almost all the resources.

b) Use of 2-3 layers of fabric is common.

c) Combining various material fabrics is also common.

d) The materials and design for the adult and pediatric masks recommended in all the resources is the same. The only difference in these types of masks is the size.

e) Limited surveys have been conducted for obtaining people's opinions on what household materials they would like to use.[19]

For easier delineation of the different literature we chose to divide the materials into categories. Note that this categorization was not material specific. Cotton, the most common household fiber was split up into clothing, bedding and towels. Synthetics tend to have a wide set of uses and compositions and were categorized by fiber type. The remaining fabrics, that sporadically appear in literature were given a separate category and any combination of the material types is separated as well. When two different fabric types were combined, that was marked as a separate category as well. The figures for dry and droplet filtration efficiency are shown in Figs A and B, respectively.

Material selection discussed in the main article were based on the entries contained in Figs A and B, latest WHO guidelines [20], and popularity of certain fabrics amongst users [19].

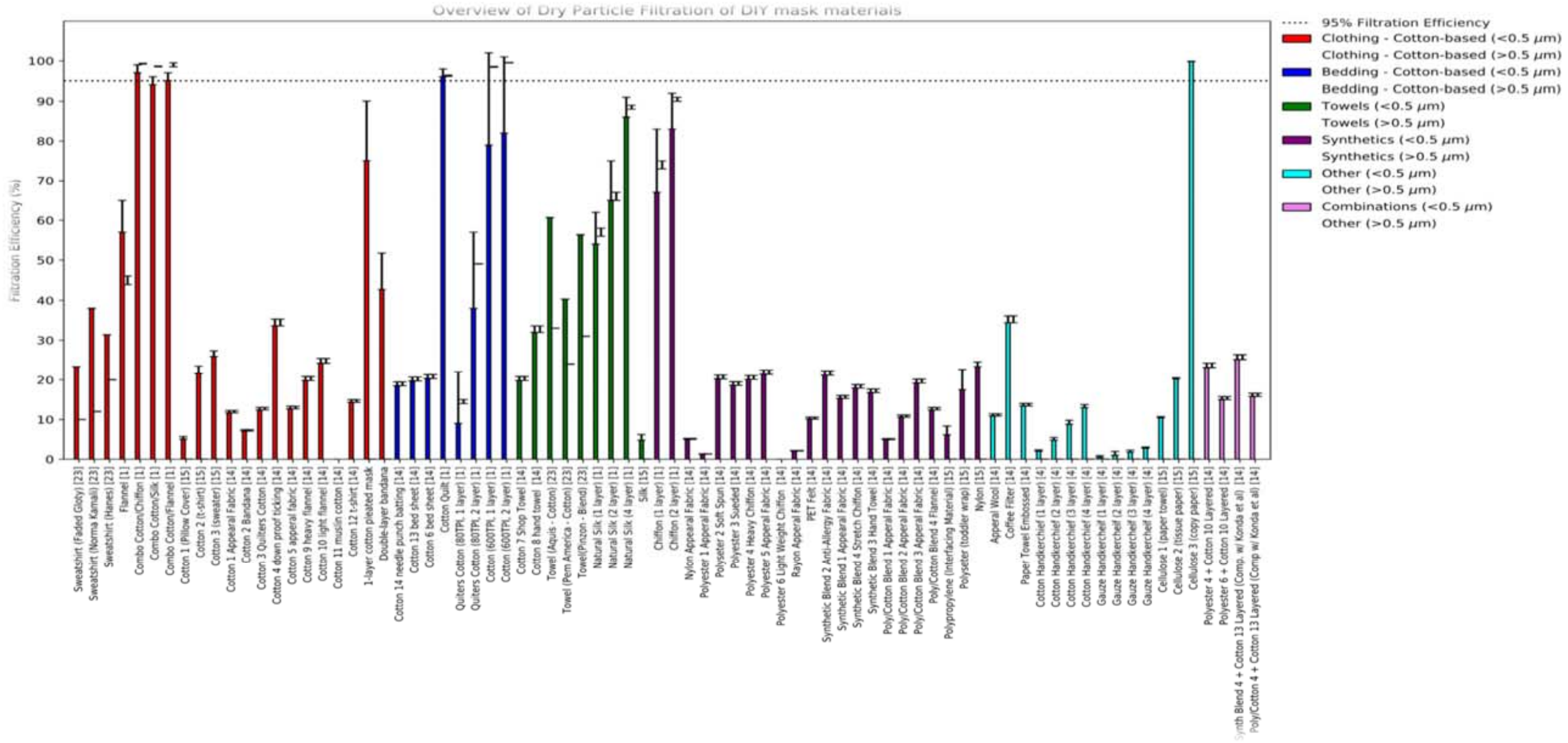

Fig A: Selected literature data on dry particle filtration efficiency of common materials. The materials are separated into categories for sake of comparison. The particle sizes were separated into < 0.5μm and > 0.5μm bins, indicated by the solid and opaque bars, respectively. The average filtration efficiency for each material category are shown as large, opaque overlays. Error bars are displayed where available in the literature. The dotted line indicates 95% filtration efficiency.

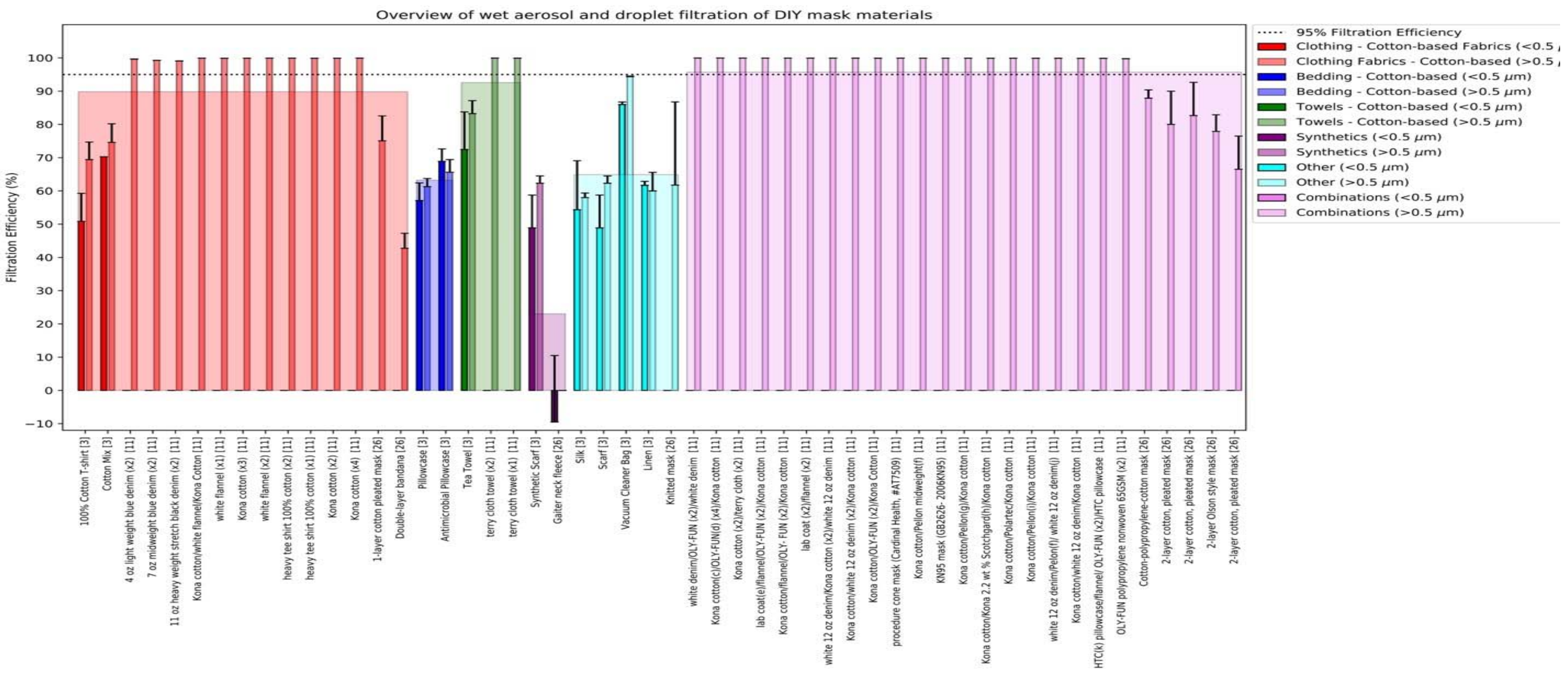

Fig B: Published values of wet/aqueous aerosol and droplet filtration efficiency of common materials. The materials are separated into categories for sake of comparison. The particle sizes studied vary and are separated into < 0.5μm and > 0.5μm bins, indicated by the solid and opaque bars, respectively. The average filtration efficiency for each material category is shown as a large, opaque overlay. Error bars are displayed where available. The dotted line indicates 95% filtration efficiency. The data here is not all inclusive. Data from one group [5] was converted from fractional transmission using the percentage of 1000 incident particles which penetrated as FE.

Microscopic structure of some fabrics used

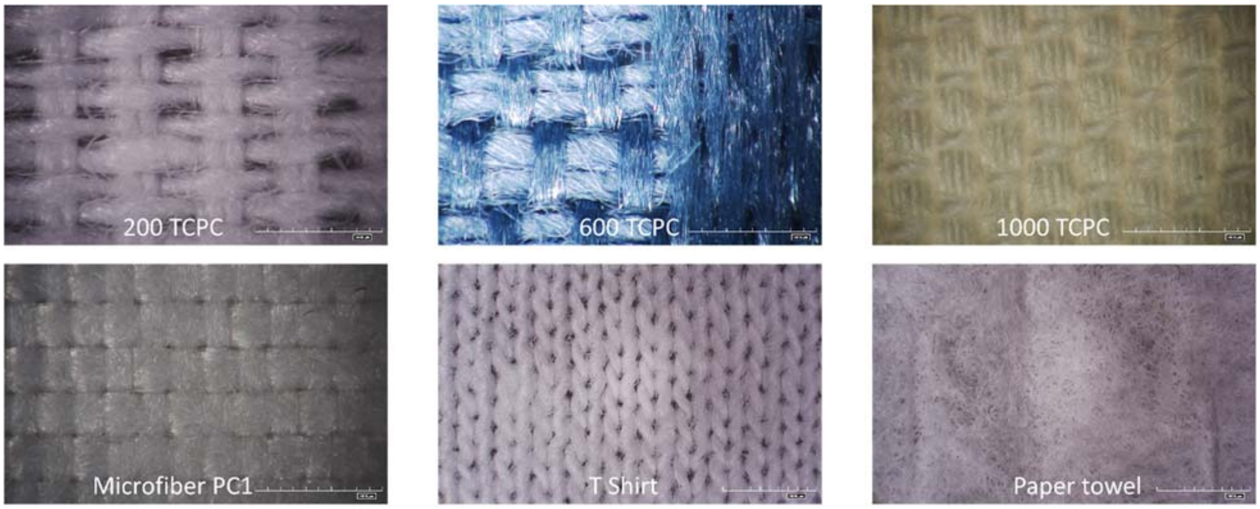

Fig C. Images from a Hirox RH-2000 Digital Microscope (Hackensack, NJ) with an MXB-2500REZ lens. Characterization was performed using at least at two magnifications, 35X and 100X. Micrographs of some woven (200 TCPC, 600 TCPC and Microfiber PC1), tightly woven (1000 TCPC), knit (T-shirt), and non-woven cellulose-based material (Paper towel) are shown here. Scale shown 2000 µm.

Experimental set up for measuring filtration efficiency of household materials against dry and solid sodium chloride aerosols

Calculations of Face Velocity

The velocity of aerosols through a filter material strongly dictates the filtration efficiency of the material. Therefore, face velocity needs be considered carefully. NIOSH recommends a flow rate of 85 L/minute rather than a specific velocity. In order to convert flow rate into a relevant velocity for fabric testing, we measured the surface area of three different brands of NIOSH certified and FDA cleared N95 respirators. One is intended for those with smaller heads, one for large, and one not specifically intended for small or large heads. The exposed surface area for three different models varied from 101.25 cm^2 to 173.5 cm^2. Converting L/minute to cm^3/s, and dividing by these areas results in a velocity that ranges from $\frac{85\times16.67}{101.25}$ cm/s = 8.3 cm/s to $\frac{85\times16.67}{173.5}$ cm/s = 14 cm/s. Pressure drop measurements were made in the small chamber used for measuring the filtration efficiency of fabrics. The plate that held the fabrics had a 4 cm by 3 cm opening, created using laser cutting. The fabric coupon was pasted onto a plate such that only a 4 cm by 3 cm piece of material was exposed. To ensure that the velocity through the fabrics was 9 cm/s, a flow rate of 6.5 L/minute was used, and all the flow passed through the fabric covering the opening.

Schematic of Experimental Set up

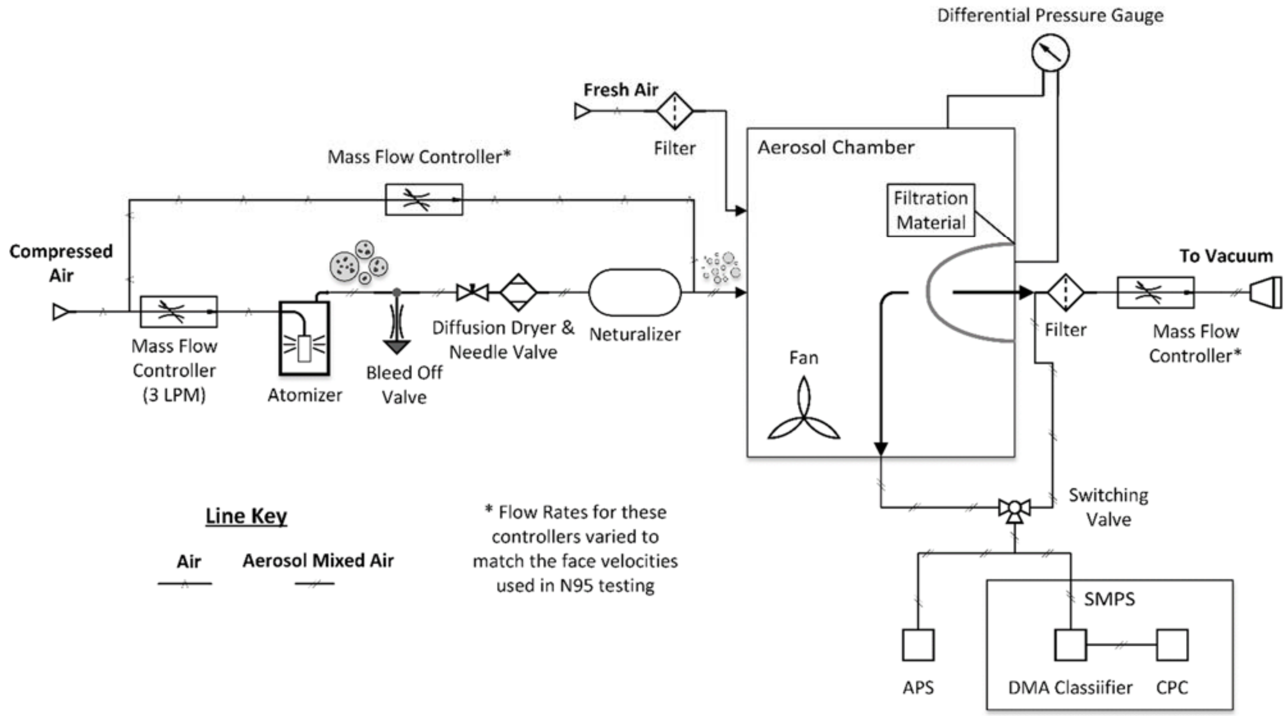

Fig D. Schematic of the experimental set up used for dry filtration efficiency testing of different fabric materials using the Scanning Mobility Particle Sizer (SMPS). Limited number of experiments were performed with the APS but those are not discussed for the purpose of this article. The pressure gauges have not been shown for clarity. The two pressure gauges with different ranges (0-6 mmH_2O and 0 – 51 mmH_20) were placed such that they could monitor the pressure drop simultaneously. The face velocity was kept constant across all experiments.

Steady state time, sampling bias, and size distribution for dried, and charge neutralized solid sodium chloride aerosols

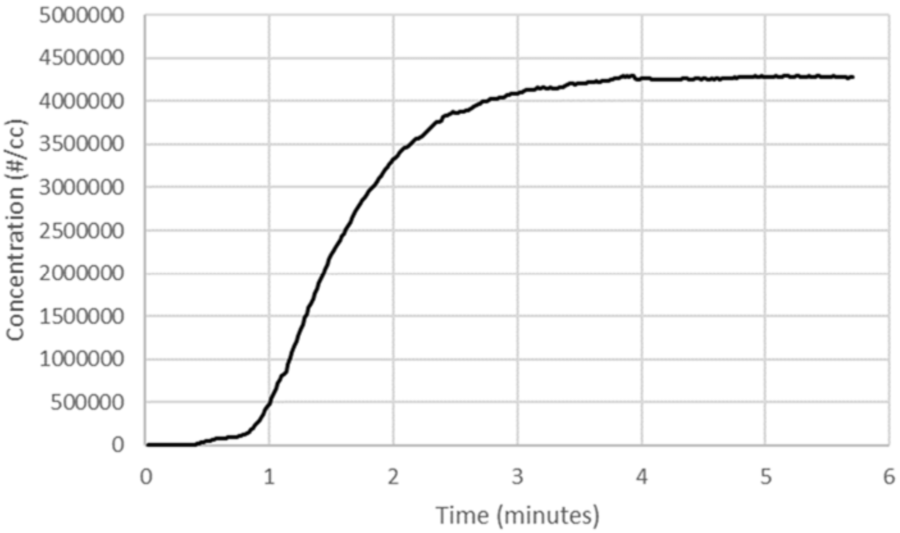

Fig E. Steady State time inside aerosol chamber. An example of concentration increases as a function of time inside the chamber obtained using a condensation particle counter (Model 3775, TSI Inc.). SMPS scans would be obtained after steady state in concentration was reached. Two chambers were used, and the same set of calibration and validation experiments (e.g. time for steady state, testing with N95 respirators) were performed for both chambers. The measurements shown here are from the upstream sampling port.

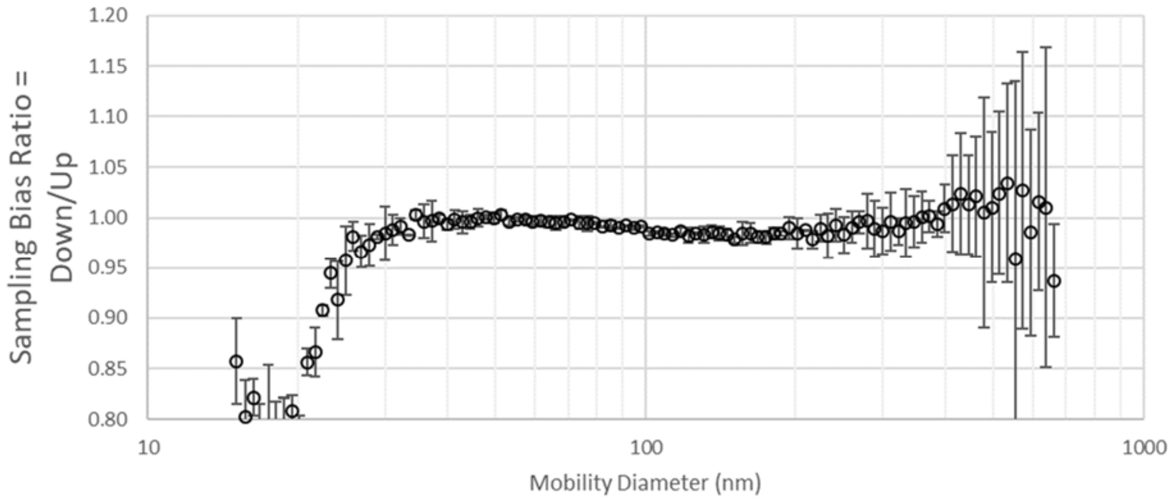

Fig F. Downstream by upstream concentration ratio. The average ratio across all sizes obtained by dividing the total downstream concentration with the upstream concentration and was 0.99. The size dependent bias was found to be within 0.95 to 1.05 for 20 nm to 660 nm. Therefore, no bias correction was performed during analysis. However, the slight biasing and inherent variations in concentration of aerosols generated from run to run resulted in some penetration values that exceeded 100 % (i.e. more particle downstream of fabric than upstream which is physically not possible), i.e. filtration efficiency that would be below 0 %. For averaging of penetration and filtration efficiency, such values were eliminated.

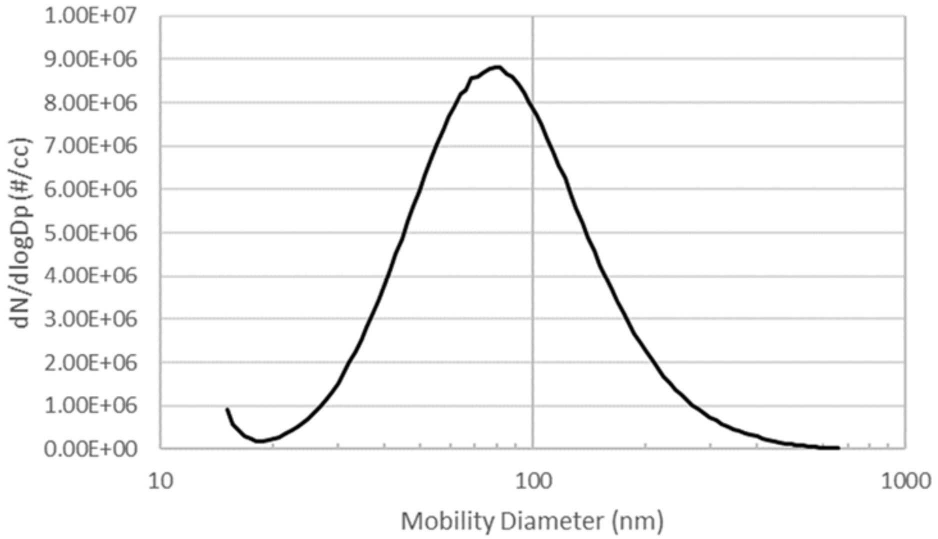

Fig G. The size distribution of the sodium chloride aerosols generated. The mean diameter of the NaCl aerosols was approximately 92.6 nm calculated using the following equation: $d_{mean} = \frac{\sum_{i=20\ nm}^{i=498.5\ nm} N \times d}{\sum_{i=20\ nm}^{i=498.5\ nm} N}$ where N is the concentration at a specific bin size, and d is the corresponding bin size.

Validation experiments with N95 respirators, and penetration efficiency of various fabrics with size

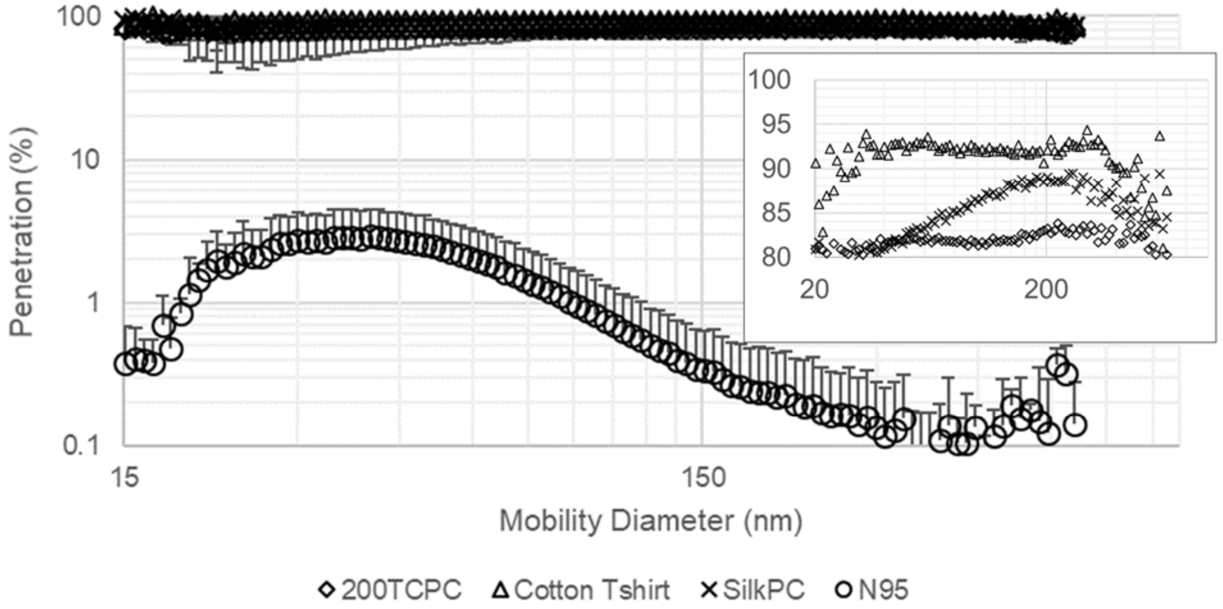

Fig H. The Penetration % of four different materials, including N95s used as controls. The high filtration efficiency materials showed the same inverted U-shaped penetration as has been reported by us and others, [21-23] and its shape is dictated by a combination of particle capture mechanisms - diffusion, impaction, interception, and electrostatics. The maximum penetration particle size (MPPS) for most modern day N95s appear at < 100 nm, because one of the middle electret layers enhances particle capture. The penetration for the fabrics plotted in the inset (without error bars for clarity) shows an inverted U shape, much like mechanical filters. The penetration increases, plateaus around 200 nm and subsequently either starts going down or plateaus out. The maximum penetrating particle size (MPPS) for these fabrics appear to be at > 200 nm. The low salt concentration at larger sizes makes it difficult to discern the trend beyond 500 nm

Mechanism of particle capture by 1000 TCPC

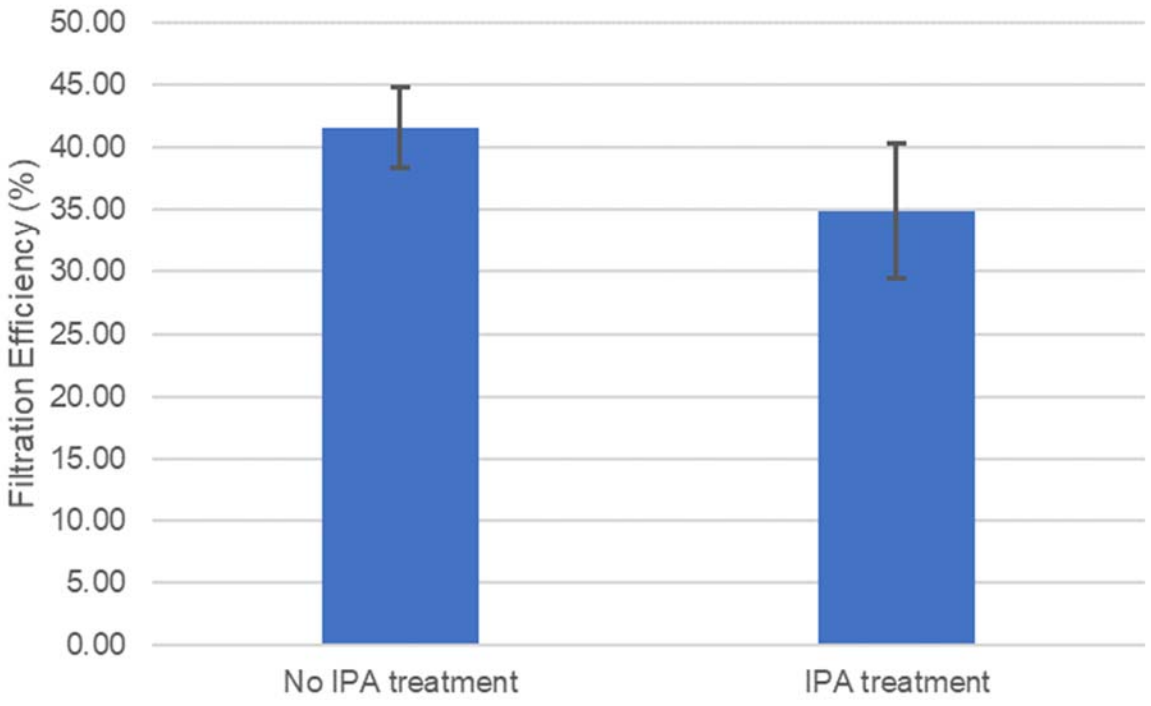

Fig I. Effect of Isopropanol (IPA) treatment on tightly woven fabric. This experiment was performed to confirm that the primary mode of particle capture is not electrostatic in nature. The 1000 TCPC fabric was dipped in IPA for 5 minutes. When N95s or surgical masks are treated with IPA, the electret filters significantly loose charge, causing a significant reduction in filtration efficiency. Unlike N95s, the filtration efficiency for IPA treated 1000 TCPC was not found to be statistically different from non-treated 1000 TCPC (p value 0.17) implying particle capture in tightly woven fabrics is likely mechanical in nature.

Impact of salt concentration on measurement of filtration efficiency of fabric

For some tightly woven fabrics we observed a trend which set them apart from other fabrics. When using 1 % NaCl concentration, the pressure drop would increase rapidly and exceed the limit of the digital pressure gauges (50.8 mm of H20). In those samples, the size-based filtration efficiency obtained with SMPS would exceed 90 %. Replacing the SMPS with a particle counter, we monitored the concentration of the aerosols upstream and downstream of the chamber and found that the downstream concentration first increases (as the chamber fills up with aerosols), but then subsequently decreases. The concentration decrease is accompanied by an increase in pressure drop. Upon switching to the upstream sampling port, no time-dependent changes in concentration were observed. This implied that the sodium chloride was starting to clog the fabric resulting in an artificial inflation of the filtration efficiency.

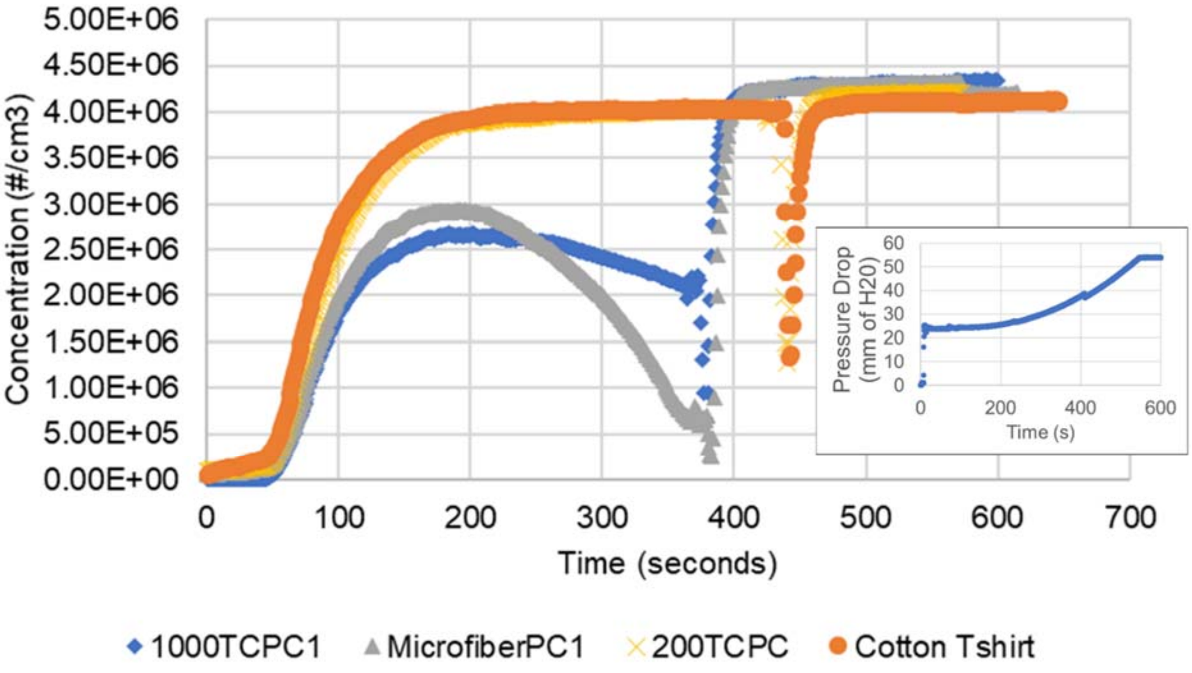

Fig J. Demonstration of the correlation between reduced aerosol concentration and increased pressure drop, for the 1000 TCPC and MicrofiberPC1 fabric. This effect was not seen in fabrics that were not very tightly knit, as evident for 200TCPC and cotton T-shirt samples. The inset shows the corresponding pressure drop increase in the 1000 TCPC fabric sample implying that the fabric was starting to load up. For the tightly woven samples, the penetration efficiency was measured by dividing the average concentration obtained downstream after steady state but before the concentration started to decline with the upstream concentration obtained when switching to the upstream port, which would remain constant throughout. The sudden dip in the concentration is attributed to the instance when the valves in the sampling ports are switched from downstream to upstream. Because the rate at which the fabrics load up is faster than the scan rate of the SMPS we use, for the tightly woven fabrics we did not obtain SMPS measurements. Furthermore, a few experiments were also performed at 0.025 – 0.05 % NaCl concentration for some tightly woven fabrics (data not shown), and the phenomenon of the fabrics starting to clog up with NaCl was not observed in those cases. This implies that salt concentration should be carefully determined when testing filtration efficiency of some fabrics. Digital gauges that can monitor pressure would also be beneficial for such tests because of their ability to track and record the real time monitoring of pressure at a rapid rate (> 1 Hz).

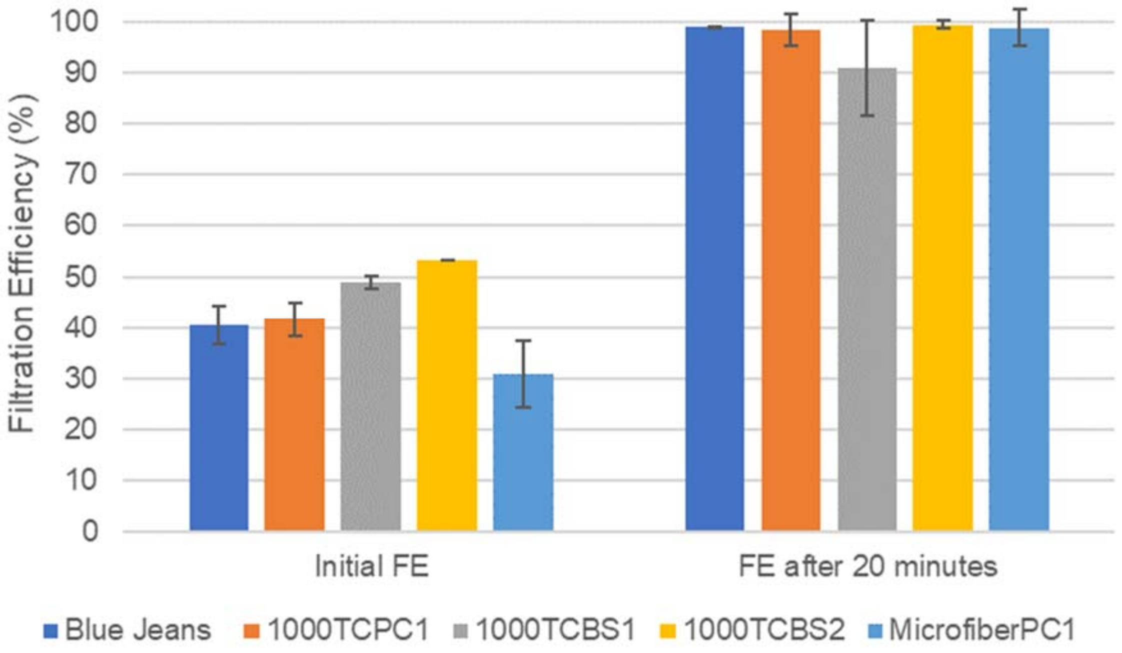

Fig K. Artificial inflation of some filtration efficiency in tightly woven fabrics. This effect may result in an inflation of efficiency by a factor of 2 to 2.5 times as seen for five different fabrics.

Comparison of dry FE with relevant literature

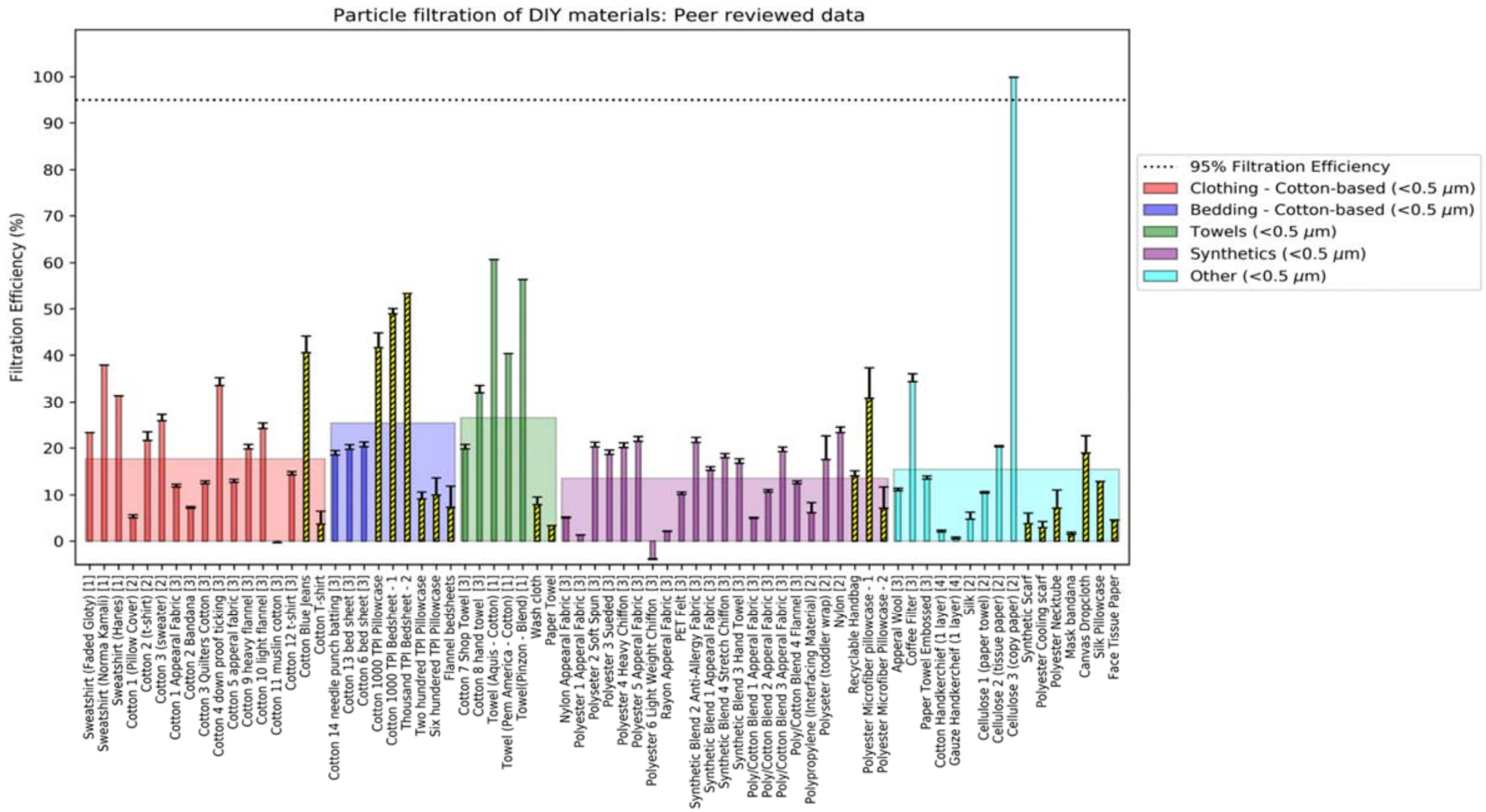

Fig L. Data obtained here (hatched yellow) is plotted against peer reviewed literature that used dried and charge neutralized sodium chloride aerosols.[3, 22, 23] One peer reviewed reference [3] was not included as it likely had unresolved errors or leaks in experimental set up as we [24] and others have pointed out via multiple letter to editors. The household materials have been categorized into various categories such as clothing, bedding etc. The average value in each category is also shown with a respective color shade. The dotted line on top represents 95 % filtration efficiency. Non peer reviewed references appearing in preprints at the time of writing this manuscript, or in websites have not been plotted for brevity.

Schematics and Some Additional Experimental Results for Water Droplets

Schematic of set up for measuring droplet filtration efficiency at high velocity

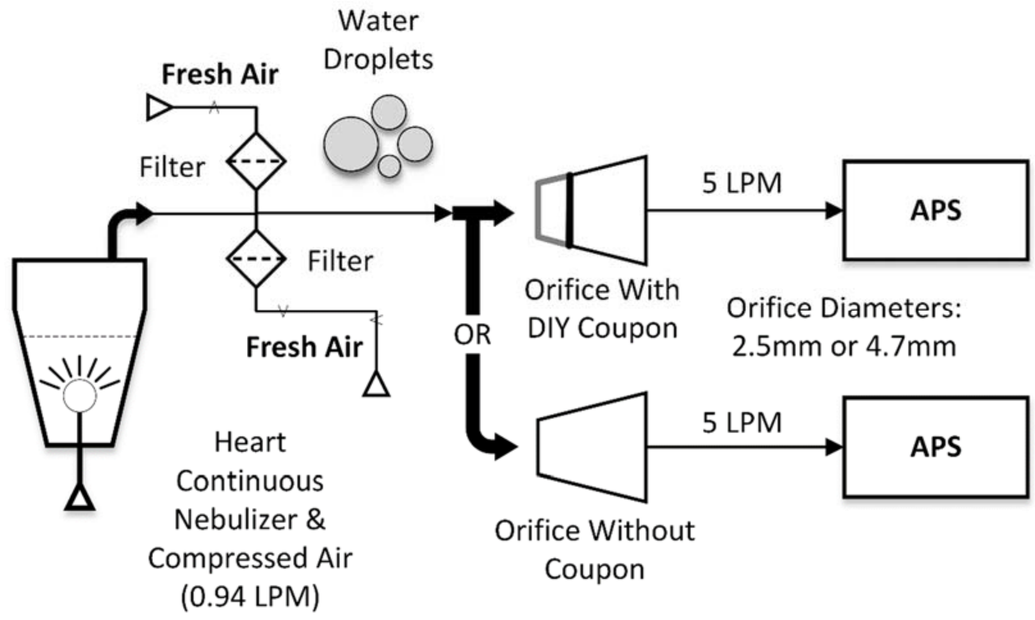

Fig M. Experimental set up used for characterizing fabric performance against droplets. (A) Schematic of the experimental set up for determining droplet filtration efficiency. The APS was operated either without the sample every 15 s for 60 s, or with the fabric coupon for every 15 seconds for 120 seconds. With an orifice diameter of 0.47 cm, and the APS pulling air through the fabric at 5 L/minute the velocity through the fabric equals = $5 \times 16.67 / (\pi \times 0.47^2 / 4) = 481$ cm/s. For a limited number of cases, the orifice diameter was further reduced to 0.25 cm to obtain velocity of 1700 cm/s. The size distribution of the droplets generated by the Heart Continuous Nebulizer. The mean aerodynamic diameter was calculated to be 3.4 µm. Unlike the collision nebulizer that generates droplets that rarely exceed 2-3 µm, this inhaler type nebulizer can generate aerosols that are bigger in size and are thus likely to be representative of cough droplets.

Impact of droplet drying during transit from fabric holder to the APS inlet port

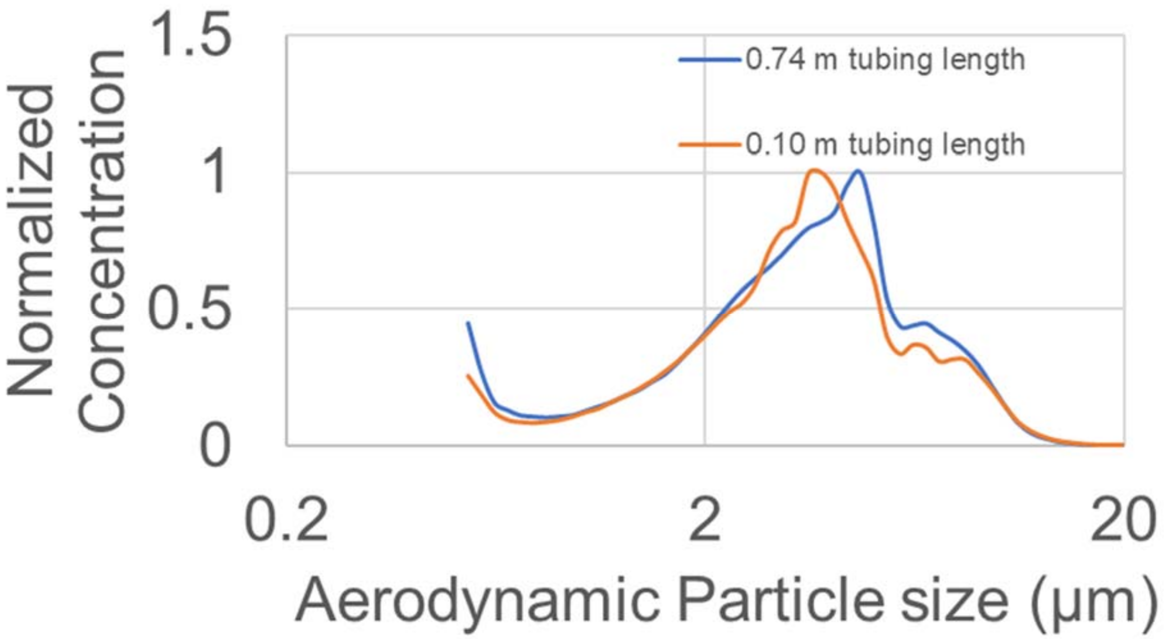

Fig N. Characterization of the water droplet diameters, and impact of location of the fabric holder relative to the APS inlet port. The tubing length here refers to the distance from where the orifice ends to the inlet of the APS in Fig M. The water droplets can evaporate and become smaller during the time they travel from the fabric holder to the APS inlet. In order to gauge the impact on the droplet size from this drying, experiments were conducted with two tubing lengths (0.1 m and 0.74 m) from the fabric holder in Fig M to the APS inlet port. While we did find water droplet concentration reduce with increasing tube length, the size of the droplets from the fabric holder to the inlet of the APS was found to remain unchanged (3.85 µm at 0.1 m versus 3.90 µm at 0.742 m). Therefore, we assumed that the droplets that impinge on the fabrics are roughly the same size as that of those that reach the APS inlet port.

Impact on the sizing accuracy of the APS at different sub-atmospheric pressures

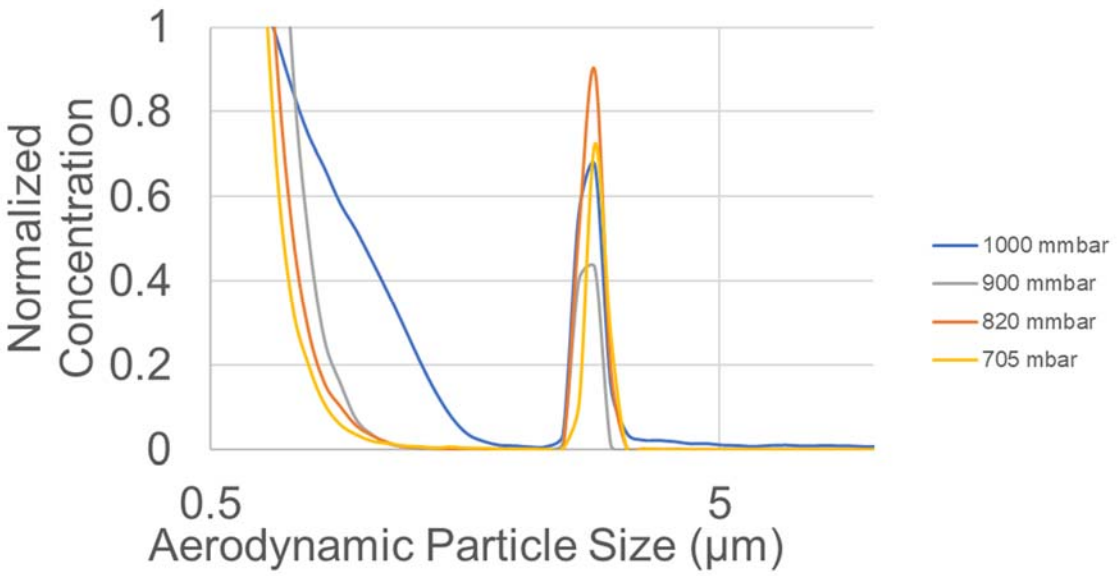

Fig O. Accuracy of the APS in characterizing size remains unimpacted at low pressure. Experiments for droplet filtration efficiencies performed at high velocities, such as the ones reported in main article, resulted in significant increase in pressure drop in the APS. Since it is not known how the low pressure (although for a relatively short time) may impact APS accuracy for size and concentration, a set of experiments was conducted in the APS that sampled and characterized 3 µm NIST traceable polystyrene latex beads. The pressure drop in the APS was controlled by a needle valve to obtain pressure ranging from 700 millibars to 1000 millibars, which covered the entire spectrum of pressure drops encountered by the fabrics we tested. The peak for the PSLs appear at approximately 2.839 µm.

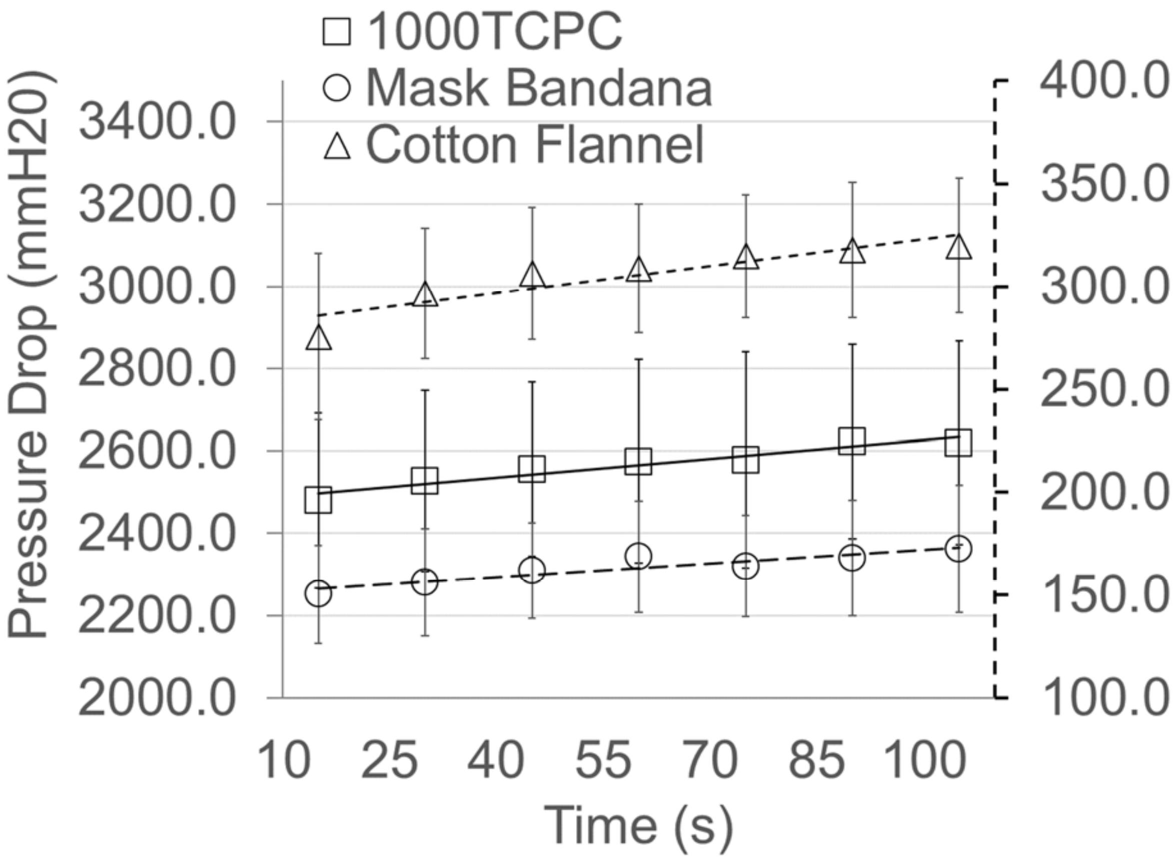

Fig P. Pressure drop across fabrics during high velocity droplet experiments. As mentioned previously, the APS also records the absolute pressure at the time it is making measurements. By taking the difference of pressure with or without fabrics we can determine the total pressure drop across each fabric which is plotted for three different fabrics as a function of time for several scans. The 1000 TCPC and mask bandana velocities in the examples plotted here was 1700 cm/s, whereas that of cotton flannel was 480 cm/s. The pressure drop increases with time likely because of water build up in the fabric.

Literature data specifically performed with droplets and comparison with findings reported here

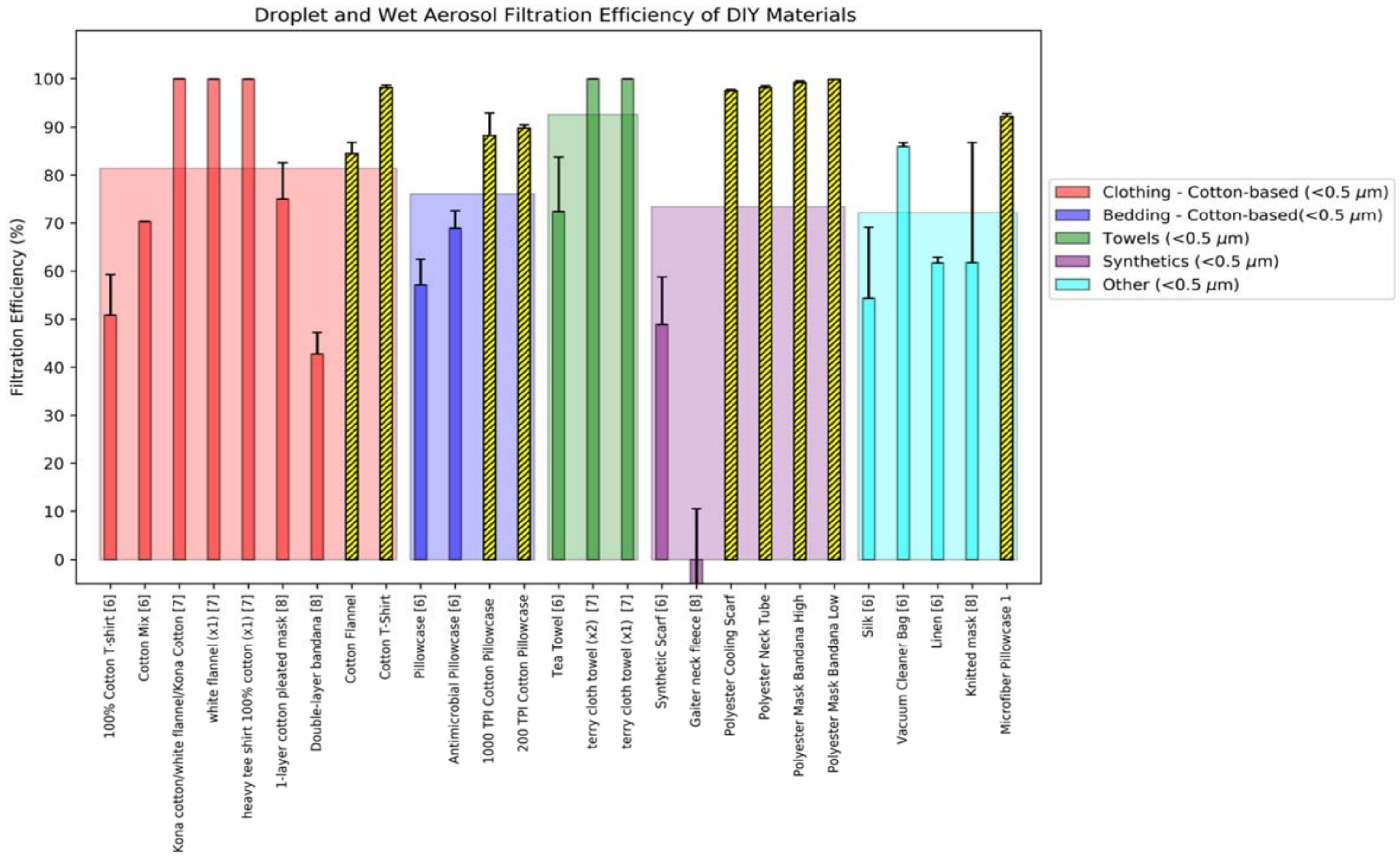

Fig Q. Data reported in the main article (hatched yellow) is plotted alongside those from other studies performed with liquid droplets. [1, 2, 5] The velocities used in each of these references varied. One used an ASTM standard, while two others relied on fluorescence (rhodamine markers) or visualization technique.

Hydrophobicity: Contact angle measurements for some fabrics

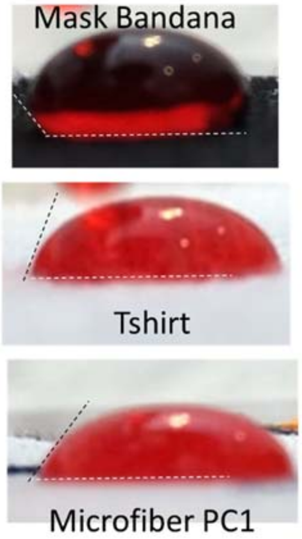

Fig R. Contact Angle Measurements. Images taken when 0.1 mL of Biotene fluid was added to the fabric. The contact angle for the fluid is time-dependent, as the angle dynamically changes as the fluid gets absorbed by the material. Therefore, for consistency across fabrics, calculations for the contact angle were made when majority (≥ 90 %) of the Biotene drop had been placed on the fabric but before the fluid would start getting absorbed by the fabric. The contact angle of Mask Bandana was measured to be 127 degrees in ImageJ (shown by the dotted lines). For T-shirt and Microfiber PC1 they appear at < 90 degrees.

Fomite, nose drop and splatter-related experiments

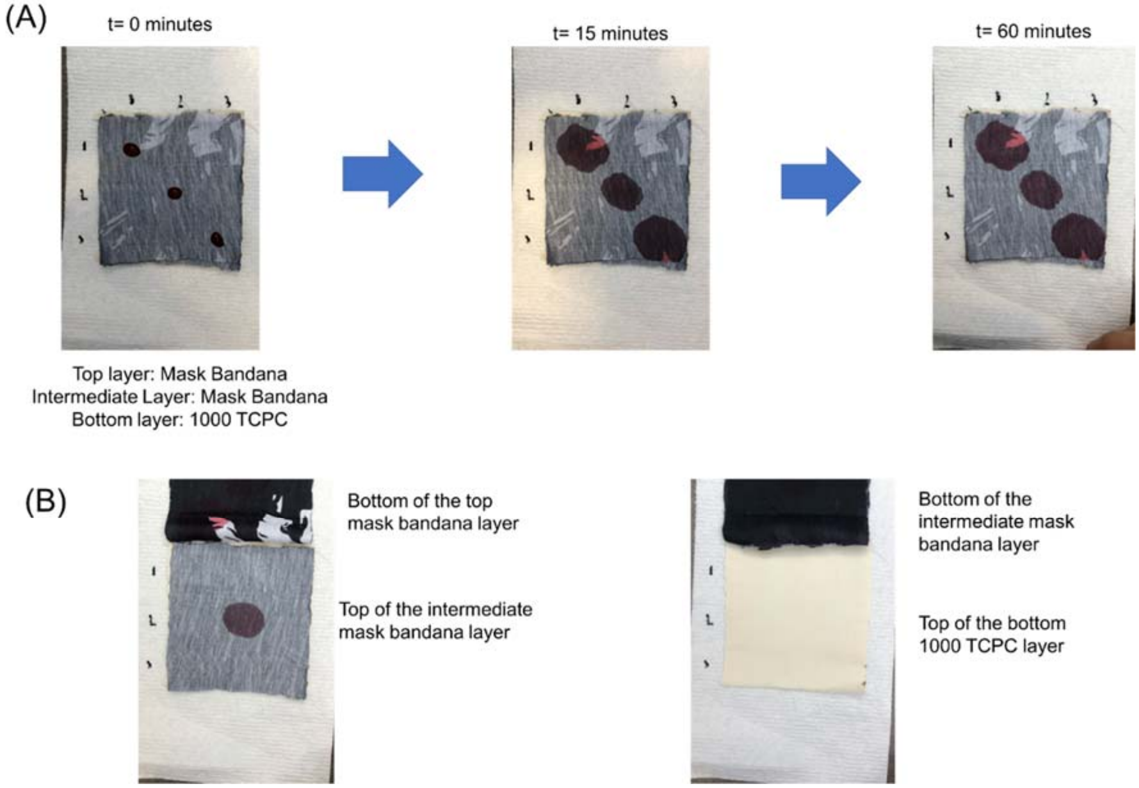

Fig S. Permeability of mucus mimicking fluid across multilayered fabrics. (A) An example permeability test is shown with one mask bandana layer on top, one intermediate mask bandana layer, and then one thousand TPI (1000 TCPC) cotton coupon at bottom. The test is monitored for at least one hour. Three time points - after addition of three drops of the saliva mimicking fluid (t=0 minutes), 15 minutes after, and 60 minutes after are shown. At the conclusion of the test, the layers were peeled while visually inspecting for the red dyed fluid. (B) The bottom of the top-most mask bandana layer, and top of the intermediate mask bandana layer. From the picture, it appears that two drops did not soak the intermediate layer, whereas a third drop did. Then the intermediate layer would be peeled to visually inspect the bottommost layer. In this case, no red dyed drops are observed on the 1000 TCPC layer, and therefore one can conclude that the combination of mask bandana – mask bandana – 1000 TCPC (not boiled) would pass the permeability test. The results from various combination materials are shown in Table 2 of the main manuscript.

Table A. Biotene permeability test for few materials. Single layer materials typically fail the permeability test. An exception is polypropylene. While all the 2 and 3 layered mask bandana – 1000 TCPC passed permeability test preboiling (12 out of 12 passes), post boiling 11 out of 12 passed (with one fail for 10-minute sample). Thus, we conclude that boiling did not appear to significantly impact the permeability of the 1000 TCPC-mask bandana layers. Refer to Table 2 in the main article for samples that were not boiled.

Inner Layer - Middle Layer(s) - Outer Layer	Permeability Test (Pass/Total Tests)
1000 TCPC - No Middle layer(s) - No Outer Layer	0/3
1000 TCPC - No Middle layer(s) - 1000 TCPC	0/3
Mask Bandana - No Middle layer(s) - No Outer Layer	0/3
Mask Bandana - No Middle layer(s) - Mask Bandana	0/3
Microfiber PC1 - No Middle layer(s) - No Outer Layer	0/3
Microfiber PC1 - No Middle layer(s) - Microfiber PC1	0/3
Polypropylene - No Middle Layer(s) - No Outer Layer	3/3
1000 TCPC - No Middle Layer(s) - Polypropylene	3/3
1000 TCPC - Polypropylene – Polypropylene	3/3
(1000 TCPC – Mask Bandana – Mask Bandana) –10 min boiled	2/3
(1000 TCPC – Mask Bandana – Mask Bandana) –60 min boiled	3/3
(1000 TCPC – Mask Bandana – Mask Bandana - Mask Bandana) –10 min boiled	3/3
(1000 TCPC – Mask Bandana – Mask Bandana - Mask Bandana) – 60 min boiled	3/3

Impact of boiling on dry Filtration Efficiency for Selected Fabrics

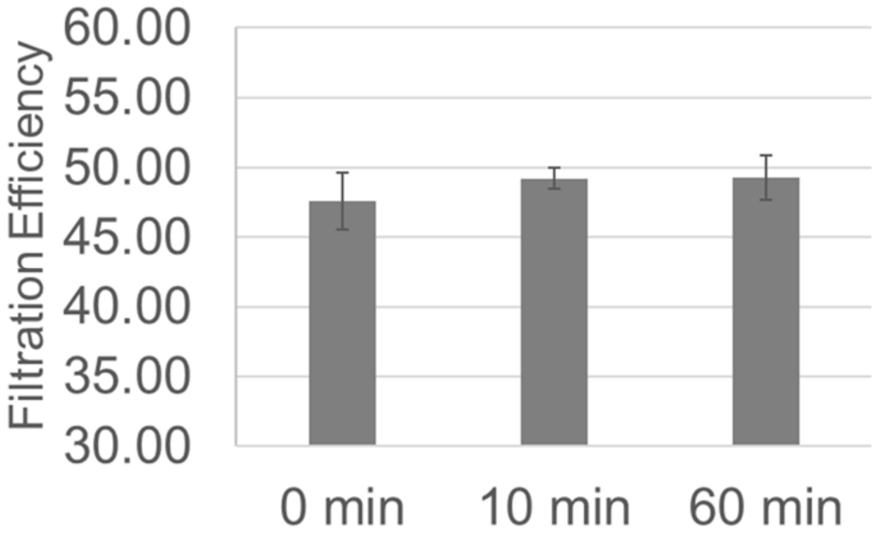

Fig T. Filtration efficiency of no-boiled, 10-minute, and 60-minute boiling. Single factor ANOVA did not reveal any statistical difference (p value = 0.507).

Supplementary References

1. Davies A, Thompson K-A, Giri K, Kafatos G, Walker J, Bennett A. Testing the Efficacy of Homemade Masks: Would They Protect in an Influenza Pandemic? . Disaster Medicine and Public Health Preparedness. 2013;7(4):413-8.
2. Fischer EP, Fischer MC, Grass D, Henrion I, Warren WS, Westman E. Low-cost measurement of facemask efficacy for filtering expelled droplets during speech. Science Advances. 2020.
3. Jung H, Jongbo KK, Lee S, Lee J, Kim J, Tsai P, et al. Comparison of Filtration Efficiency and Pressure Drop in Anti-Yellow Sand Masks, Quarantine Masks, Medical Masks, General Masks, and Handkerchiefs. Aerosol and Air Quality Research. 2014;14(3):991-1002.
4. Konda A, Prakash A, Moss GA, Schmoldt M, Grant GD, Supratik. G. Aerosol Filtration Efficiency of Common Fabrics Used in Respiratory Cloth Masks. ACS Nano. 2020;14(5):6339-47.
5. Lustig SR, Biswakarma JJH, Rana D, Tilford SH, Hu W, Su M, et al. Effectiveness of Common Fabrics to Block Aqueous Aerosols of Virus-like Nanoparticles. ACS Nano. 2020;14(6):7651-8.
6. Onur A, Emon MAB, Cheng S, Liu; H, Chamarro LP, Saif MTA. Performance of fabrics for home-made masks against spread of respiratory infection through droplets: a quantitative mechanistic study. Extreme Mechanics Letters. 2020;40.
7. Rengasamy S, Eimer B, Shaffer RE. Simple Respiratory Protection—Evaluation of the Filtration Performance of Cloth Masks and Common Fabric Materials Against 20–1000 nm Size Particles. Annals of Work Exposures and Health. 2010;54(7):789-98.
8. Rodriguez-Palacios A, Cominelli F, Basson AR, Pizarro TT, Ilic S. Textile Masks and Surface Covers—A Spray Simulation Method and a “Universal Droplet Reduction Model” Against Respiratory Pandemics. Frontiers in Medicine. 2020;7:260.
9. Zangmeister C, Radney J, Vicenzi E, Weaver J. Filtration Efficiencies of Nanoscale Aerosols by Cloth Mask Materials Used to Slow the Spread of SARS CoV-2. ACS Nano. 2020.
10. Zhao M, Lia L, Xioa W, Yu X, Wang H, Wang Q, et al. Household Materials Selection for Homemade Cloth Face Coverings and Their Filtration Efficiency Enhancement with Triboelectric Charging. Nano Letters. 2020;20(7):5544-52.
11. Nationwide Children. Why You Shouldn’t Mask a Baby. [Available from: https://www.nationwidechildrens.org/family-resources-education/700childrens/2020/04/mask-safety-101]
12. Daughters CsHoTKs. Mask Safety for Children. 2020 [Available from: https://www.chkd.org/Blog/Mask-Safety-for-Children/.
13. VerywellFamily. Face Masks and Kids: How to Make a Mask Your Kids Will Wear. 2020 [Available from: https://www.verywellfamily.com/how-to-make-masks-for-your-family-4802120.
14. Great Falls Clinic Guidelines. Approved pattern info for homemade masks. 2020. [Available from: https://www.gfclinic.com/approved-pattern-info-for-homemade-masks/]
15. University of Utah Health. Homemade Cloth Face Masks. 2020. [Available from: https://healthcare.utah.edu/healthfeed/postings/2020/04/homemade-cloth-face-masks_web.pdf]
16. Children’s Hospital Minnesota 2020 [Available from: https://www.childrensmn.org/2020/04/07/help-prevent-the-spread-of-covid-19-how-to-make-homemade-face-masks/.

17. World Health Organization. Advice on the use of masks for children in the community in the context of COVID-19 2020 [Available from: https://apps.who.int/iris/bitstream/handle/10665/333919/WHO-2019-nCoV-IPC_Masks-Children-2020.1-eng.pdf.
18. Children's Hospital of Philadelphia. Making Homemade Face Masks. 2020. [Available from: https://www.chop.edu/how-make-homemade-diy-face-mask]
19. SmartAir. The Ultimate Guide to Homemade Face Masks for Coronavirus 2020 [Available from: https://smartairfilters.com/en/blog/best-diy-coronavirus-homemade-mask-material-covid/.
20. World Health Organization. Advice on the use of masks in the context of COVID-19 2020 [Available from: https://www.who.int/publications/i/item/advice-on-the-use-of-masks-in-the-community-during-home-care-and-in-healthcare-settings-in-the-context-of-the-novel-coronavirus-(2019-ncov)-outbreak.
21. Guha S, Mejía-Alfaro A, Hariharan P, Myers MR. Effectiveness of facemasks for pediatric populations against submicron-sized aerosols. American Journal of Infection Control. 2015;43(8):871-7.
22. Zangmeister C, Radney J, Vicenzi E, J W. Filtration Efficiencies of Nanoscale Aerosols by Cloth Mask Materials Used to Slow the Spread of SARS CoV-2. ACS Nano. 2020.
23. Zhao M, Lia L, Xioa W, Yu X, Wang H, Qang Q, et al. Household Materials Selection for Homemade Cloth Face Coverings and Their Filtration Efficiency Enhancement with Triboelectric Charging. Nano Letters. 2020;20(7):5544-52.
24. Carr IA, P; Guha, S. Letter to the Editor Regarding Aerosol Filtration Efficiency of Common Fabrics Used in Respiratory Cloth Masks. ACS Nano. 2020;14(9):10754–5.
